# *Plasmodium vivax* Transmission in Africa

**DOI:** 10.1371/journal.pntd.0004222

**Published:** 2015-11-20

**Authors:** Rosalind E. Howes, Robert C. Reiner Jr., Katherine E. Battle, Joshua Longbottom, Bonnie Mappin, Dariya Ordanovich, Andrew J. Tatem, Chris Drakeley, Peter W. Gething, Peter A. Zimmerman, David L. Smith, Simon I. Hay

**Affiliations:** 1 Spatial Ecology and Epidemiology Group, Department of Zoology, University of Oxford, Oxford, United Kingdom; 2 Center for Global Health and Diseases, Case Western Reserve University, Cleveland, Ohio, United States of America; 3 Department of Epidemiology and Biostatistics, School of Public Health, Indiana University, Bloomington, Indiana, United States of America; 4 Fogarty International Center, National Institutes of Health, Bethesda, Maryland, United States of America; 5 Wellcome Trust Centre for Human Genetics, University of Oxford, Oxford, United Kingdom; 6 Department of Geography and Environment, University of Southampton, Highfield, Southampton, United Kingdom; 7 Flowminder Foundation, Stockholm, Sweden; 8 Malaria Centre, London School of Hygiene and Tropical Medicine, London, United Kingdom; 9 Sanaria Institute for Global Health and Tropical Medicine, Rockville, Maryland, United States of America; 10 Institute for Health Metrics and Evaluation, University of Washington, Seattle, United States of America; Barcelona Centre for International Health Research (CRESIB) and Institució Catalana de Recerca i Estudis Avançats (ICREA), SPAIN

## Abstract

Malaria in sub-Saharan Africa has historically been almost exclusively attributed to *Plasmodium falciparum* (*Pf*). Current diagnostic and surveillance systems in much of sub-Saharan Africa are not designed to identify or report non-*Pf* human malaria infections accurately, resulting in a dearth of routine epidemiological data about their significance. The high prevalence of Duffy negativity provided a rationale for excluding the possibility of *Plasmodium vivax* (*Pv*) transmission. However, review of varied evidence sources including traveller infections, community prevalence surveys, local clinical case reports, entomological and serological studies contradicts this viewpoint. Here, these data reports are weighted in a unified framework to reflect the strength of evidence of indigenous *Pv* transmission in terms of diagnostic specificity, size of individual reports and corroboration between evidence sources. Direct evidence was reported from 21 of the 47 malaria-endemic countries studied, while 42 countries were attributed with infections of visiting travellers. Overall, moderate to conclusive evidence of transmission was available from 18 countries, distributed across all parts of the continent. Approximately 86.6 million Duffy positive hosts were at risk of infection in Africa in 2015. Analysis of the mechanisms sustaining *Pv* transmission across this continent of low frequency of susceptible hosts found that reports of *Pv* prevalence were consistent with transmission being potentially limited to Duffy positive populations. Finally, reports of apparent Duffy-independent transmission are discussed. While *Pv* is evidently not a major malaria parasite across most of sub-Saharan Africa, the evidence presented here highlights its widespread low-level endemicity. An increased awareness of *Pv* as a potential malaria parasite, coupled with policy shifts towards species-specific diagnostics and reporting, will allow a robust assessment of the public health significance of *Pv*, as well as the other neglected non-*Pf* parasites, which are currently invisible to most public health authorities in Africa, but which can cause severe clinical illness and require specific control interventions.

## Author Summary

*Plasmodium vivax* (*Pv*) is the most widely distributed malaria parasite globally, but conspicuously “absent” from Africa. The majority of African populations do not express the Duffy blood group antigen, which is the only known receptor for *Pv* infection. Since this discovery in the 1970s, the low clinical incidence of *Pv* in Africa has resulted in a perception of *Pv* being completely absent and any apparent cases being misdiagnoses, and no public health allowances are made for this parasite in terms of diagnosis, treatment or surveillance reporting. As more sensitive diagnostics become available, *Pv* infection in Africa is increasingly reported from a variety of different survey types: entomological, serological, community prevalence surveys, as well as clinical infection data from local residents and travellers returning to malaria-free countries. A literature review was conducted to assemble these reports and assess the current status of evidence about *Pv* transmission in Africa. Moderate to conclusive evidence of transmission was available from 18 of the 47 malaria-endemic countries examined, distributed across all parts of the continent. Mechanisms explaining this reported transmission are evaluated, as well as alternative explanations for the observations. Combinations of complementary explanations are likely, varying according to regional ecology and population characteristics. The public health implications of these observations and recommendations for increased awareness of *Pv* transmission on this continent are discussed.

## Introduction

Malaria in sub-Saharan Africa has historically been almost exclusively attributed to *Plasmodium falciparum* (*Pf*). The identification of the Duffy antigen as the obligate trans-membrane receptor for *P*. *vivax* (*Pv*) infection of red blood cells by Miller *et al*. during the 1970s [1,2] stalled research into the epidemiology of *Pv* in Africa as indigenous populations on this continent were known to rarely express the Duffy antigen (and therefore be resistant to infection) and the dogma of “*Pv* absence from Africa” became entrenched [3,4]. However, against a backdrop of increasing appreciation for the clinical severity of *Pv* infection [5–7], multiple sources of evidence suggest that *Pv* may be more prevalent on this continent than commonly perceived [3,8–10]. The absence of any thorough effort to unify these sporadic reports, however, precludes appraisal of their significance, and assessment of the public health significance of *Pv* in sub-Saharan Africa.

*Plasmodium vivax* has certain biological and epidemiological characteristics distinguishing it from *Pf*. Lower peripheral parasitaemia of blood-stage infections and a perception of lower risk of clinical complications during the era of malariotherapy of neuro-syphilis patients [5,11] contributed to the classification of *Pv* as “benign”, despite the parasite causing the same spectrum of clinical symptoms as *Pf* [5,7,12,13]. A key difference is the *Pv* parasite’s ability to form dormant liver-stages (“hypnozoites”) which evade the human immune system and blood-stage therapy, and can trigger relapses of clinical episodes weeks to months following the initial infective mosquito bite [14,15]. Without adequate treatment, hypnozoites therefore impose on the host a cumulative burden of blood-stage infection, contributing to the disease’s severe malaria phenotype, notably as severe anaemia [16]. These (and other) life-stage features unique to *Pv* mean that several aspects of the control interventions designed against *Pf*, including preventive measures related to vector biting behaviour [17,18], diagnosis and treatment [15,19,20] are not effective against *Pv*, therefore making this parasite a greater challenge than *Pf* in achieving malaria elimination [21,22]. Current WHO diagnosis and treatment guidelines for the WHO African Region (AFR) do not allow for the peculiarities of *Pv* infections, and are exclusively focused on *Pf* (see Box 1). Untreated, however, chronic relapses of *Pv* can cause severe morbidity and mortality [16].

**Box 1. Current WHO Guidelines towards *Pv* in WHO African Region (AFR)**

Information is taken from the World Malaria Report (WHO, 2014 [29]); Guidelines for the Treatment of Malaria (WHO, 2015 [30]); Universal Access to Malaria Diagnostic Testing: An Operational Manual (WHO, 2011 updated in 2013 [84]); and Good Practices for Selecting and Procuring Rapid Diagnostic tests for Malaria (WHO, 2011 [85]).

**Diagnosis**

Since 2010, WHO policy is that all suspected malaria cases must be promptly diagnosed by microscopy or rapid diagnostic test (RDT). An estimated 62% of suspected cases were tested across AFR in 2013. However, ensuring high quality microscopy is not feasible at all health-care levels. Instead, RDT use is being scaled-up, and their circulation has doubled since 2010 and accounted for 52% of all diagnoses in 2013.

AFR countries (except for Eritrea and Ethiopia) are recommended to use RDTs that detect only *Pf*. These tests have generally higher thermal stability and the *Pf*-specific antigen HRP2 has highest sensitivity. In the Horn of Africa, combination RDTs using both HRP2 for detecting *Pf* and antigens for detecting non-*Pf* infections are recommended. Varied antigens are used to detect *Pv*: either exclusively (pLDH-*Pv*) or as a non-*Pf* infection (pLDH-*Pvom*) or as an unspecific *Plasmodium* infection (pLDH-*pan*). An infection can only be confirmed as *Pv* if the RDT includes the pLDH-*Pv* antigen. Only 5 countries report using RDTs that include *Pv*-specific diagnostic capacity (pLDH-*Pv*): Angola, Eritrea, Madagascar, Tanzania, Sudan. Seven countries report the Pf + other (pLDH-*Pvom*) RDT combination: Comoros, DRC, Gabon, Namibia, Nigeria, Rwanda, South Sudan. Eleven use *Pf*-only RDTs (HRP2), and the remainder of countries (23) did not report their RDT policy to the WMR in 2014.

Furthermore, RDT product testing demonstrates that only 42% of *Pv*-specific RDT tests have acceptable detection scores for low parasitaemia infections, contrasting with 76% of kits for *Pf*.

**Treatment**

Radical cure for *Pv* requires a blood-stage drug and a hypnozoitocide. Chloroquine is the default, but in areas of resistance, ACTs should be used. Primaquine prevents hypnozoite-triggered relapse, but should ideally only be administered to known G6PD normal patients. In areas where G6PD testing is not available, “a decision to prescribe primaquine must be based on an assessment of the risks and benefits of adding primaquine”. Treatment without primaquine results in repeated clinical episodes and increased risk of severe morbidity, but primaquine triggers varying degrees of haemolysis in G6PD deficient individuals. *Plasmodium vivax*-specific treatment policies exist for 7 of the 45 AFR countries considered here: either chloroquine or artemisinin-based treatment combined with primaquine in most cases. For the remaining 38 countries, there is no recommended policy tailored to *Pv* treatment.

Community surveys in Liberia published in 1949 reported 2.0% of all malaria infections being due to *Pv* [23]. However, the acceptance of *P*. *ovale* (*Po*) as a separate species [24,25], first as a single species and later two [26], and the demonstration of the dependency of *Pv* on the Duffy antigen meant that the idea of *Pv* absence from Africa became entrenched, and during the latter part of the twentieth century apparent diagnoses of *Pv* were routinely reclassified as *Po* [3], a morphologically very similar parasite [24]. During this period of near-total reliance on microscopy diagnosis, *Pv* largely disappeared from sub-Saharan African epidemiological records. The rise of the molecular diagnostics era, however, provided the capacity to confidently differentiate human *Plasmodium* infections [27], and with it came the first substantive evidence bringing into doubt both the universality of Miller’s observations and the absence of *Pv* from populations across sub-Saharan Africa. The other endemic *Plasmodium* species, *Po* and *P*. *malariae* (*Pm*) have lower parasitaemia than *Pv* [28], so are also historically neglected by microscopy-based diagnostics, and therefore also rarely recorded without molecular diagnosis.

While *Pv* is evidently not a major co-endemic parasite in sub-Saharan Africa, Mendis *et al* in 2001 nevertheless estimated an annual burden of 6–15 million cases across Africa [9]. Reports of *Pv* transmission from almost all countries across the continent [8] further justify closer investigation. A shifting attitude is discernible in the language of the 2014 World Malaria Report, which suggests that *Pv* can occur throughout Africa, but with a low risk of infection due to high prevalence of Duffy negativity [29]; and the 2015 WHO Treatment Guidelines [30] indicate that cases are rare outside the Horn of Africa (with the exception of Mauritania and Mali) [30]. Current WHO guidelines for diagnosis and treatment in AFR, however, do not reflect this risk, and for example, recommend rapid diagnostic tests that do not detect non-*Pf* species. Moreover, most sub-Saharan African countries have no country-specific guidelines for *Pv* treatment (see Box 1).

The epidemiology of *Pv* in Africa remains poorly understood, despite being key to evaluating the need for adapting current control interventions, to estimating the burden of *Pv* disease globally, and to anticipating any changes that may result from reductions in *Pf* transmission [22]. Here we assess the existing evidence base of *Pv* transmission in sub-Saharan Africa and evaluate the hypothesised mechanisms which may be sustaining this.

## Methods

The broad aims of this study were to evaluate what, if any, evidence existed of *Pv* transmission in Africa, and to explore whether the small subset of known susceptible individuals (i.e. Duffy positives) could be sustaining the observed prevalence levels reported from community surveys. Here, details are given about (i) the evidence base assembly, (ii) the unified framework weighting the evidence, (iii) quantification of the Duffy positive population at risk of infection (*Pv*PAR), (iv) the prevalence data analysis in relation to *Pv*PAR, and (v) reports of *Pv* infections in Duffy negative hosts.

### Assembling an evidence base of *P*. *vivax* transmission in sub-Saharan Africa

**Fig 1:**
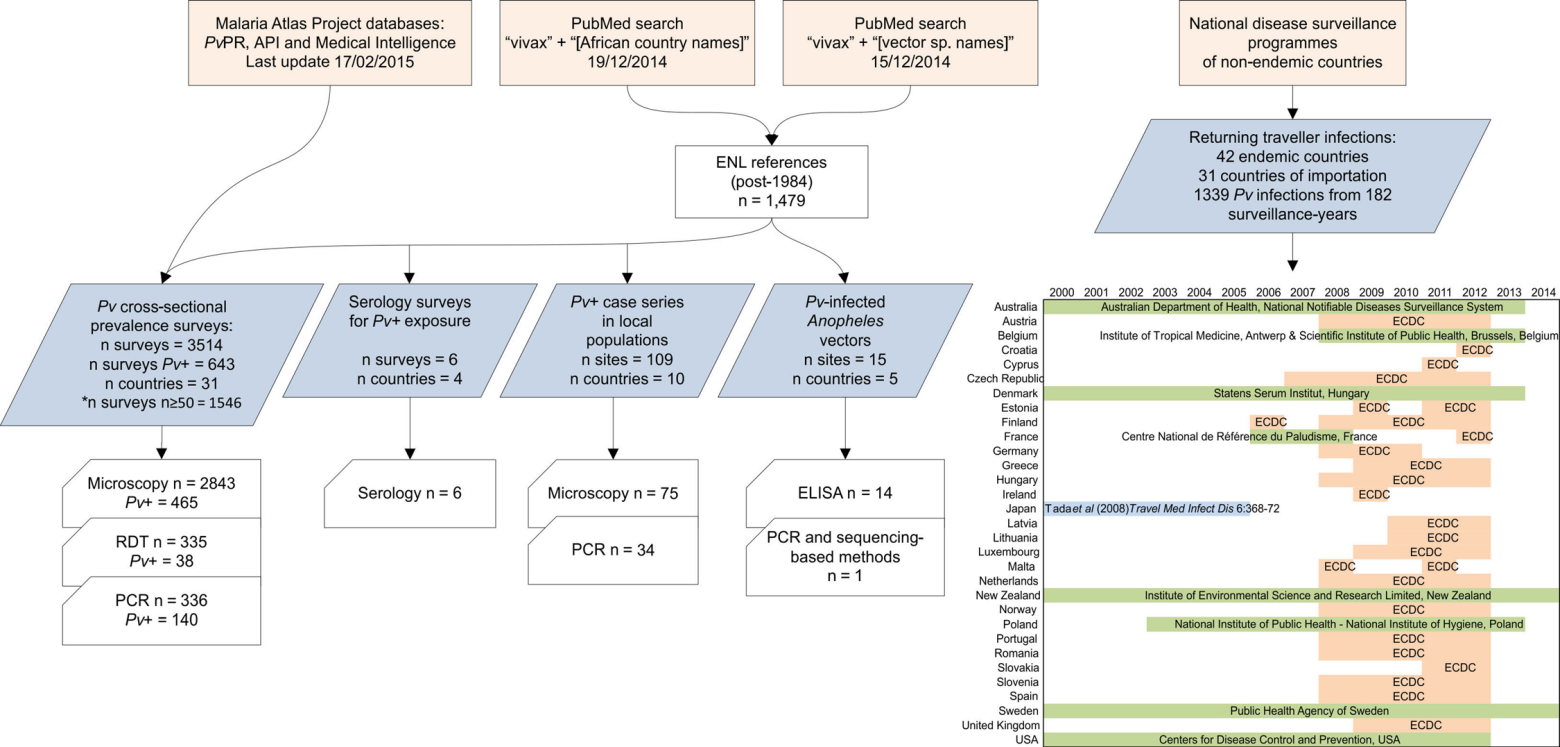
Data assembly procedure. The geographic extent of the analysis included the 47 malaria-endemic countries of sub-Saharan Africa: all WHO African Regional Office countries except Algeria (0 indigenous cases in 2013 [29]), but including Djibouti, the Republic of Sudan and Somalia. ECDC: European Centre for Disease Prevention and Control. *Prevalence survey analyses were limited to those with sample size ≥50.

A PubMed literature search starting 01/01/1985 was run using keywords “vivax” AND “[African country names] or [Africa]” (last updated 19/12/2014). Following abstract review for relevance, several categories of data emerged: locally-diagnosed clinical case reports, serological surveys, observations of infected vectors, cross-sectional prevalence surveys, and reports of imported malaria into non-endemic countries by infected travellers (Fig 1). The location, diagnostic details and numbers of *Pv* infections were recorded by data type. *Pv*-specific annual parasite incidence (API) data reported by health management information systems was also assembled (see Gething *et al* 2012 for details [31]).

A further literature search for references about African *Anopheles* vectors was run (15/12/2014), using keywords “vivax” AND “[vector species names]”, with the seven dominant vector species across the Africa region [32]: *Anopheles arabiensis*, *An*. *funestus*, *An*. *gambiae*, *An*. *melas*, *An*. *merus*, *An*. *moucheti* and *An*. *nili*; *An*. *pharoensis* was also included as a WHO-defined “major *Anopheles* species” in certain African countries [29]. The numbers of *Pv* sporozoite-positive mosquitoes found in each survey location were recorded.

The most commonly used epidemiological metric of malaria endemicity is the parasite rate (PR), assessed by cross-sectional surveys of infection prevalence [33]. The Malaria Atlas Project (www.map.ox.ac.uk) PR surveys database, the product of nearly ten years of archiving [33], was reviewed for evidence of *Pv* infections reported since 1985 (last updated 17/2/15).

A dataset of imported malaria infections into malaria-free countries by travellers returning from sub-Saharan African countries was assembled from national and regional (notably the European Centre for Disease Prevention and Control, ECDC) surveillance programme reports (search window November 2014 –January 2015). A selection of malaria-free countries with robust surveillance systems were contacted to obtain summary surveillance data since 2000 on imported malaria cases into their country. Given that individuals from these non-endemic countries would have similar prior exposure (i.e. be predominantly immunologically naïve), their country of origin was not relevant to the analysis, although varied countries were approached to increase the coverage of countries of infection (for instance, countries from several continents [Europe, Americas, Asia, Oceania] and linguistic groups [Dutch, English, French, German, Portuguese, Spanish, etc]). Contributing countries and the reporting time intervals which could be assembled are listed in Fig 1. Total numbers of infections were aggregated by probable country of infection.

### A weighted framework of the strength of reported evidence of transmission

**Fig 2:**
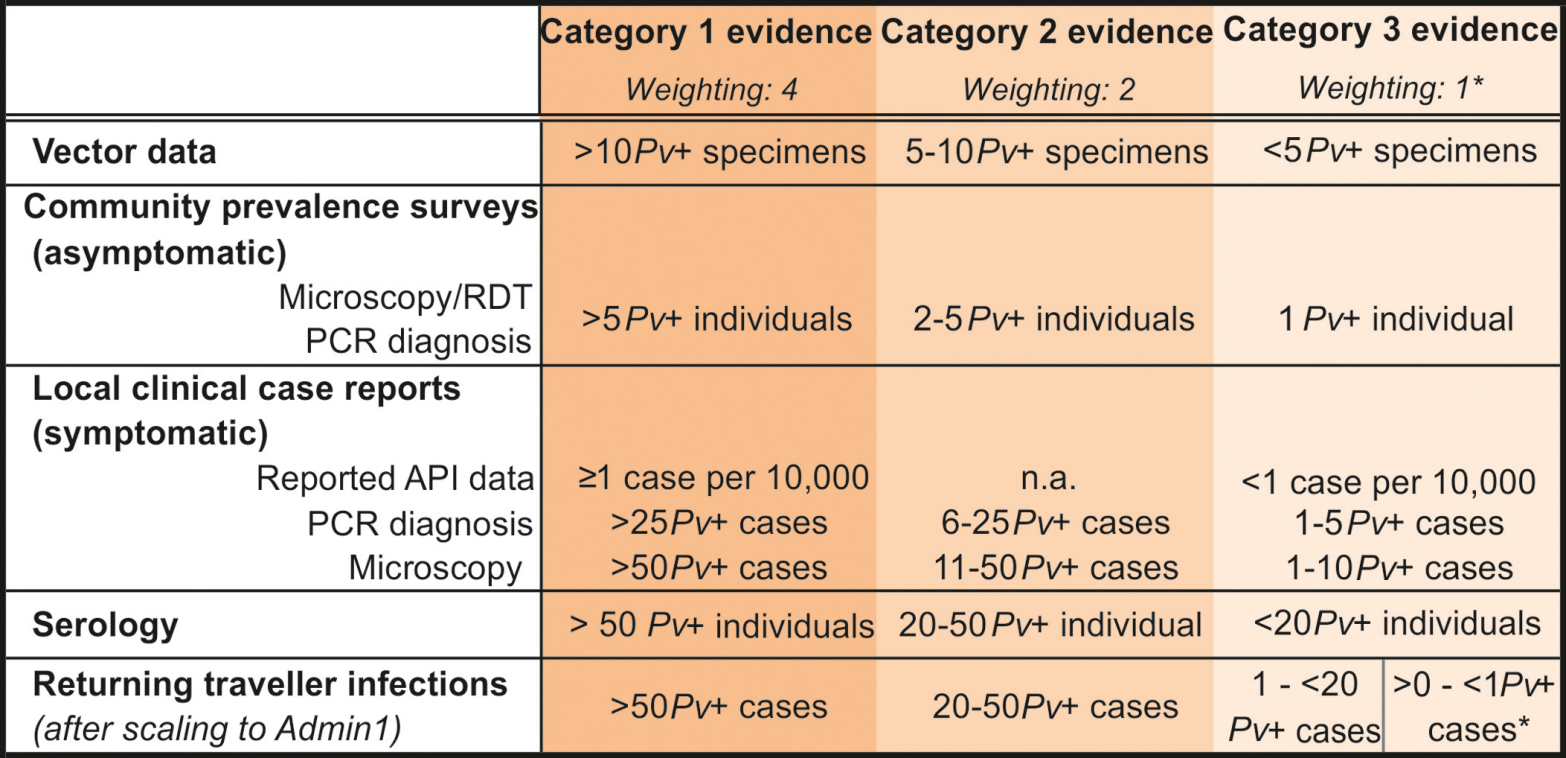
Weighting of the different data types at Admin1 level. Ranks refer to the relative strength of the evidence as an indicator of ongoing *Pv* transmission in an area. *The total number of returning traveller infections from each country was divided between the number of Admin1 regions per country. If there was less than 1 reported infection per Admin1, the weighting score was 0.5. Note that because the traveller infections data were retrospective and not sub-nationally specific, it was not possible to distinguish the Republic of Sudan from South Sudan, thus results were considered for Sudan pre-separation and the same score allocated to both countries based on the overall data.

A framework was developed to assess the overall evidence of *Pv* transmission at the first sub-national administrative level (Admin1). First, the data available for each data type from each Admin1 were classified as being of category 1 (strongest), 2, or 3 (weakest) evidence (Fig 2), and second, the scores of the seven surfaces representing each evidence type were summed to provide an overall assessment of the relative strength of evidence of *Pv* transmission occurring in each Admin1. The varied nature of the available data restricted this to being a qualitative analysis, without aiming at any quantitative estimate of endemicity/incidence or quantitative comparison with other *Plasmodium* parasites. The absolute number of positive reports was used as the metric of strength of evidence, rather than any proportional metric.

The criteria for each evidence category varied according to the time window of exposure represented by the data type (and thus the probability of local infection), the denominator size, and the diagnostic method sensitivity/specificity. For example, due to their limited range of movement, infected vector specimens were considered relatively strong evidence of local transmission. In contrast, the longer window of exposure detected by serological surveys meant sero-positivity could result from a transmission event in a different location prior to relocation to the study site. To reflect the reduced temporal specificity of the serological evidence, a relatively higher number of reports of sero-positive individuals was therefore necessary to provide the same strength of evidence of transmission as infections observed in real-time. The single time-point snapshot investigated during a community prevalence survey contrasts with a much larger time window and population denominator for clinical case reporting across a region. Therefore, evidence of parasitaemia from prevalence surveys was considered stronger evidence of ongoing transmission than routine reporting of symptomatic cases, and a larger number of positive symptomatic individuals was required to equate to the strength of evidence of asymptomatic infections from prevalence surveys. Annual Parasite Incidence data (API) indicating stable transmission (≥1 case/10,000) equated to the strongest category 1, while unstable transmission (<1 case/10,000) was category 3.

Clinical cases diagnosed by molecular diagnostic tools based on nucleic acid amplification techniques (notably PCR) were considered stronger evidence of transmission than by light microscopy or rapid diagnostic tests (RDT) due to the increased species-specific diagnostic accuracy. In the case of prevalence surveys, however, where infections were more likely to be of low parasitaemia, RDT and microscopy were expected to diagnose a smaller proportion of total infections than PCR-based diagnostics due to their differing limits of the detection [28]. It has been estimated that molecular diagnostics identify at least double the number of infections compared to conventional methods [34]. Therefore the numbers of infections diagnosed by the two methods correspond to differing proportions of the true parasite prevalence, with RDT and microscopy-based underestimating true prevalence. On the other hand, RDT and microscopy diagnostics were less specific. These two aspects therefore balanced out and it was considered that prevalence data from both diagnostics would correspond to similar strength of evidence of transmission.

Reports of *Pv* infection among travellers returning from African countries could not be attributed the same strength of evidence as observations of infection among local residents. The relapsing nature of *Pv* parasites means that infections could have been acquired from travel prior to the reported journey. Furthermore, the nature of the imported infections dataset assembled here is highly opportunistic and incomplete. This data type was therefore down-weighted relative to reports of local infections.

The data were mapped to the Admin1 level. Where multiple surveys within a data type were available from a single administrative region, the scores were based on the total number of reports. Returning traveller infections were only reported to the national level, so to allocate scores to Admin1 units, the overall number of reported *Pv* infections was divided by the total number of Admin1 units in the country. These infections were therefore strongly down-weighted relative to the other data.

Finally, once the component data types were categorised and mapped, these were summed together in ArcMap 10.1 [35]. The weighting system accounted both for the strength of evidence in each category and for corroboration between data types: the more data types reporting *Pv* transmission, the higher the overall strength of evidence. The highest level, “conclusive evidence”, was only attained if two or more category 1 sources of evidence were available. The lowest level, “very weak evidence” corresponded to Admin1 units from which no direct evidence was available, only a relatively small number of infected traveller reports (less than 1 case per number of Admin1 units, thus a low probability of local transmission).

### Estimating the population at risk of infection

Using an approach similar to that previously published [8,31], the geographic limits of *Pv* transmission were overlaid onto a 2015 population surface to estimate the *Pv*PAR. Medical intelligence data and biological exclusions were applied to define the limits of potential transmission. First, API and routinely reported incidence data were reviewed [31] to identify areas which could be excluded for being risk-free of malaria; this exclusion was not species-specific due to the previously discussed imperfect diagnostic capacity of most countries, but instead only areas defined as “malaria free” were excluded. Second, areas where temperatures could not support *Pv* sporogony at any time in an average year were excluded by a modelled temperature suitability mask [36]. Aridity was not used as exclusion given that man-made conditions can facilitate vector breeding even in areas of extreme aridity; previous *Pv*PAR estimates have “downgraded” risk from stable to unstable transmission levels based on aridity, but the lack of distinction between these levels here means no aridity exclusion was applied. Third, urban areas, as identified based on the Global Rural Urban Mapping Project (GRUMP) urban extents layer [37], were excluded. While potentially overly-conservative, the exclusion of urban populations was consistent with previous *Pv*PAR estimates [8,31], and is based on the significantly lower infection risk in urban areas [38,39].

A population surface for 2015 was compiled from the WorldPop Project projections (www.worldpop.org), supplemented by data from the Gridded Population of the World (GPW) v3 (http://sedac.ciesin.columbia.edu/data/set/gpw-v3-population-count-future-estimates) for the Comoros and São Tomé and Príncipe, which were not available from WorldPop. This was adjusted to the Duffy positive population using the previously published Duffy blood group frequency maps developed by geostatistical modelling of population surveys of blood group frequencies [4]. The median model prediction of the Duffy surface was used, together with the 25% and 75% quartiles (50% confidence interval) of the Duffy model outputs. National-level *Pv*PAR estimates were derived for the three Duffy thresholds within the defined limits of transmission. All spatial manipulations were run at 5 x 5 km resolution in ArcMap and ArcScene 10.1 [35].

### Investigation of reported *Pv*PR in relation to the Duffy positive *Pv*PAR

The PR database was used to investigate aspects of *Pv* transmission across sub-Saharan Africa and compare them to the *Pv*PAR and to *Pf*. Estimates of the prevalence of Duffy negativity at the PR survey locations were extracted from the modelled Duffy group frequency maps [4]. For the comparisons with *Pf*, only surveys with the diagnostic capacity to identify both species were included, ensuring that paired estimates of *Pv*PR and *Pf*PR were matched in space, time, and population sample characteristics. The analysis was also restricted to surveys of ≥50 individuals. A trio of spatially-matched estimates were therefore collated for *Pv*PR, *Pf*PR and Duffy negativity prevalence.

This dataset was used to assess: (i) whether the observed *Pv*PR values were consistent with infections being potentially limited exclusively to Duffy positive hosts, (ii) how the prevalence of infection and relative risk of infection differed between species among their specific subset of known susceptible hosts (based on the assumption that the *Pv*PAR was limited to Duffy positive hosts: *Pv*PR_Fy+_ = *Pv*PR/Proportion of Duffy positive hosts), (iii) the relationship between infection prevalence of the two species, and whether prevalence of one could inform predictions of the other. Full methodological details are available in Text S1.

### Collating reports of *P*. *vivax* infections in Duffy negative hosts

Reports of *Pv* infections in Duffy negative hosts were compiled from the different data types reviewed. Only studies that used molecular confirmation of both *Pv* infection and Duffy genotype were included. The reports were geopositioned and presented in relation to the local prevalence of Duffy blood group phenotypes estimated by the modelled geostatistical maps [4].

## Results

### Evidence of transmission: The data

**Fig 3:**
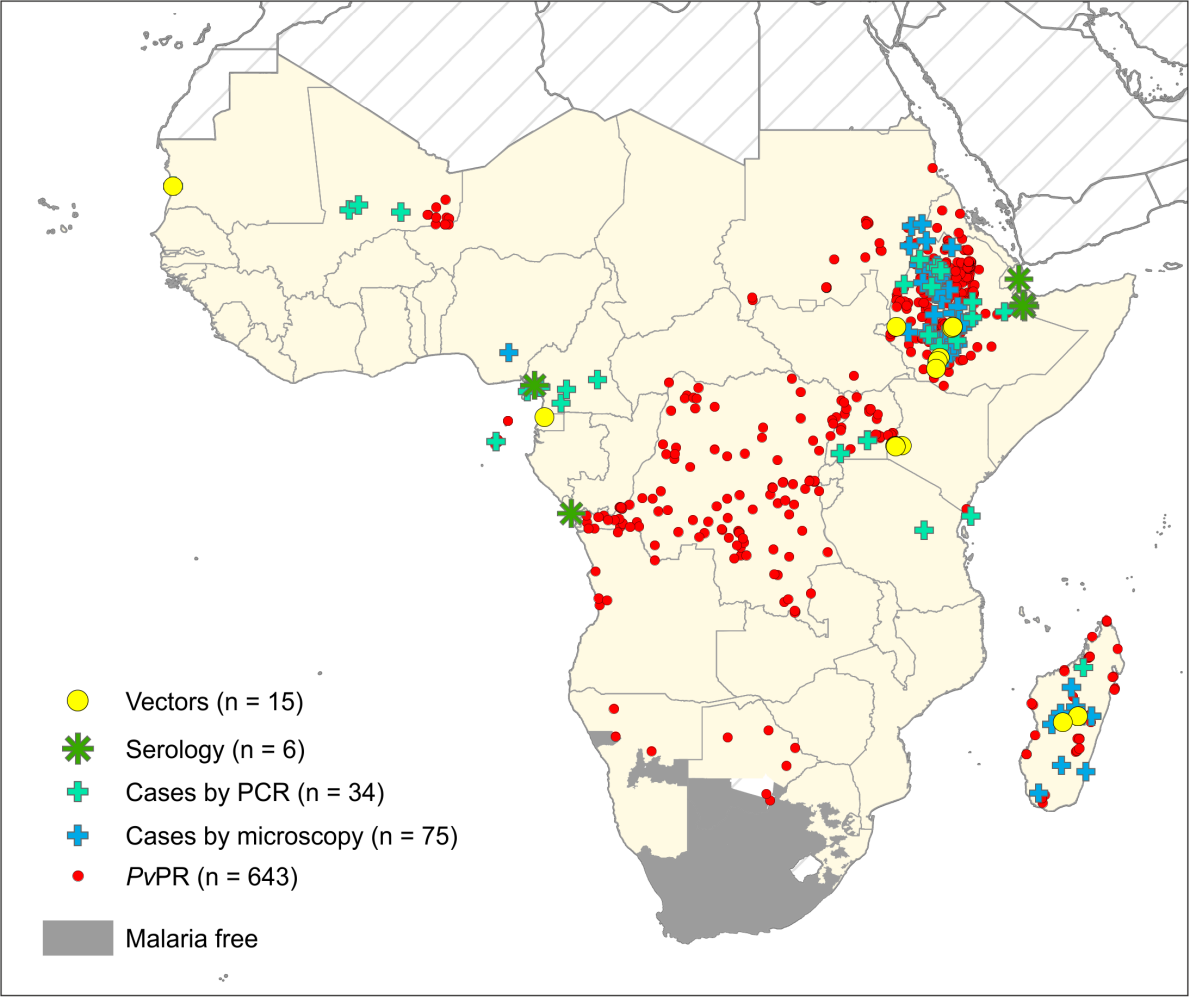
The evidence base of *Pv* occurrence in sub-Saharan Africa. All points represent a positive diagnosis of *Pv*. See S1–S3 Tables for original data.

**Fig 4:**
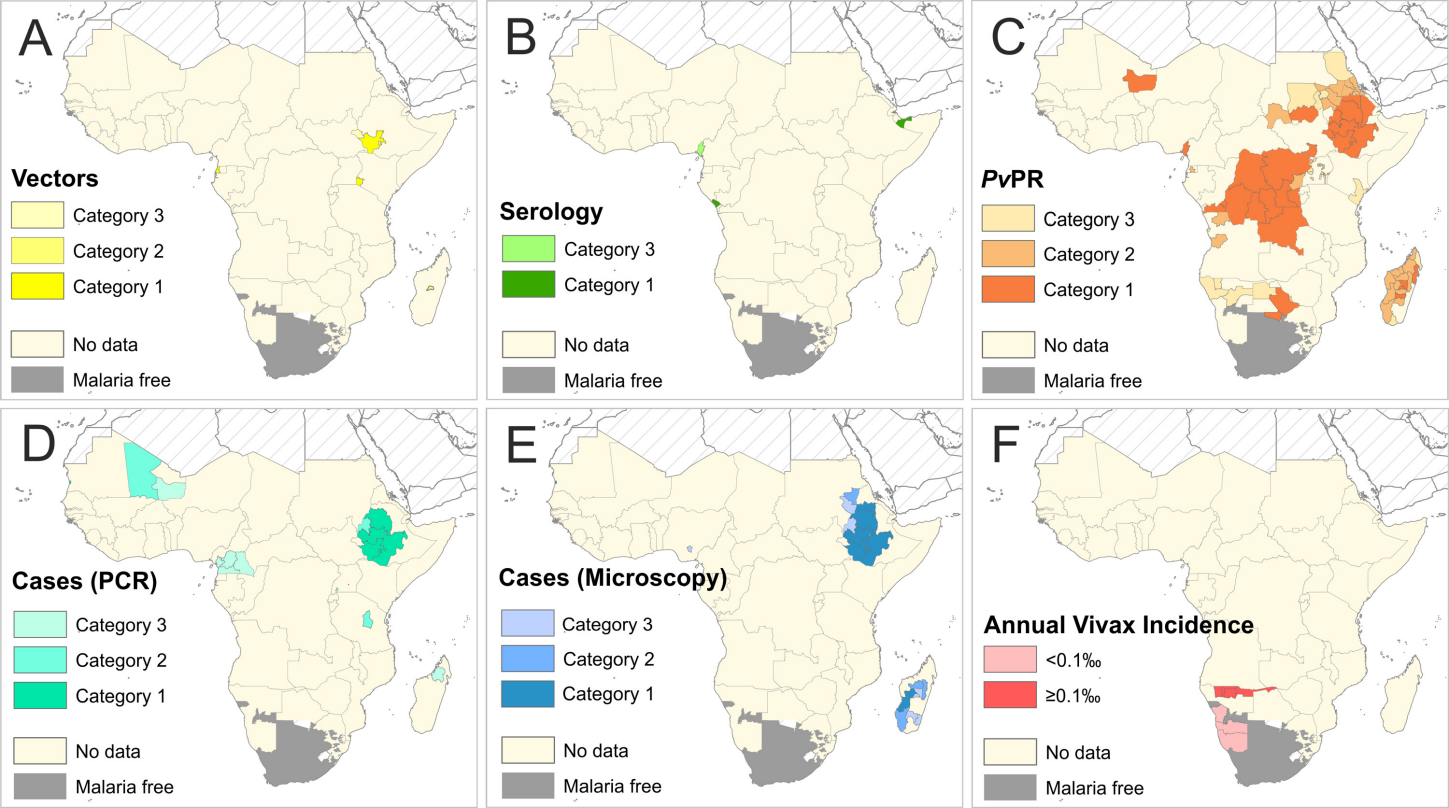
Summaries of the overall category of evidence available for each data type at the Admin1 level. Evidence categories are defined in Fig 3. Panel A represents evidence of *Pv* from infected vectors, Panel B sero-positivity, Panel C *Pv* community prevalence surveys, Panel D molecularly-diagnosed clinical cases, Panel E microscopy-diagnosed clinical cases and Panel F annual *Pv* incidence data. See S1–S3 Tables for original data.

The literature search identified 1,479 publications, of which 529 were considered relevant to studies of *Pv* in Africa. All reports of *Pv* infection were collated and geopositioned (Fig 3). A total of 15 surveys reporting infected *Anopheles* vectors were identified (S1 Table), as well as six reports of *Pv* sero-positivity (S2 Table), 109 sites with local *Pv* case reports (S3 Table) and 643 *Pv*-positive community prevalence surveys (www.map.ox.ac.uk). Overall, reports of *Pv* were available from 21 of the 47 malaria-endemic countries examined. The maps in Fig 4 represent the aggregated scores mapped to the Admin1 level.

**Fig 5:**
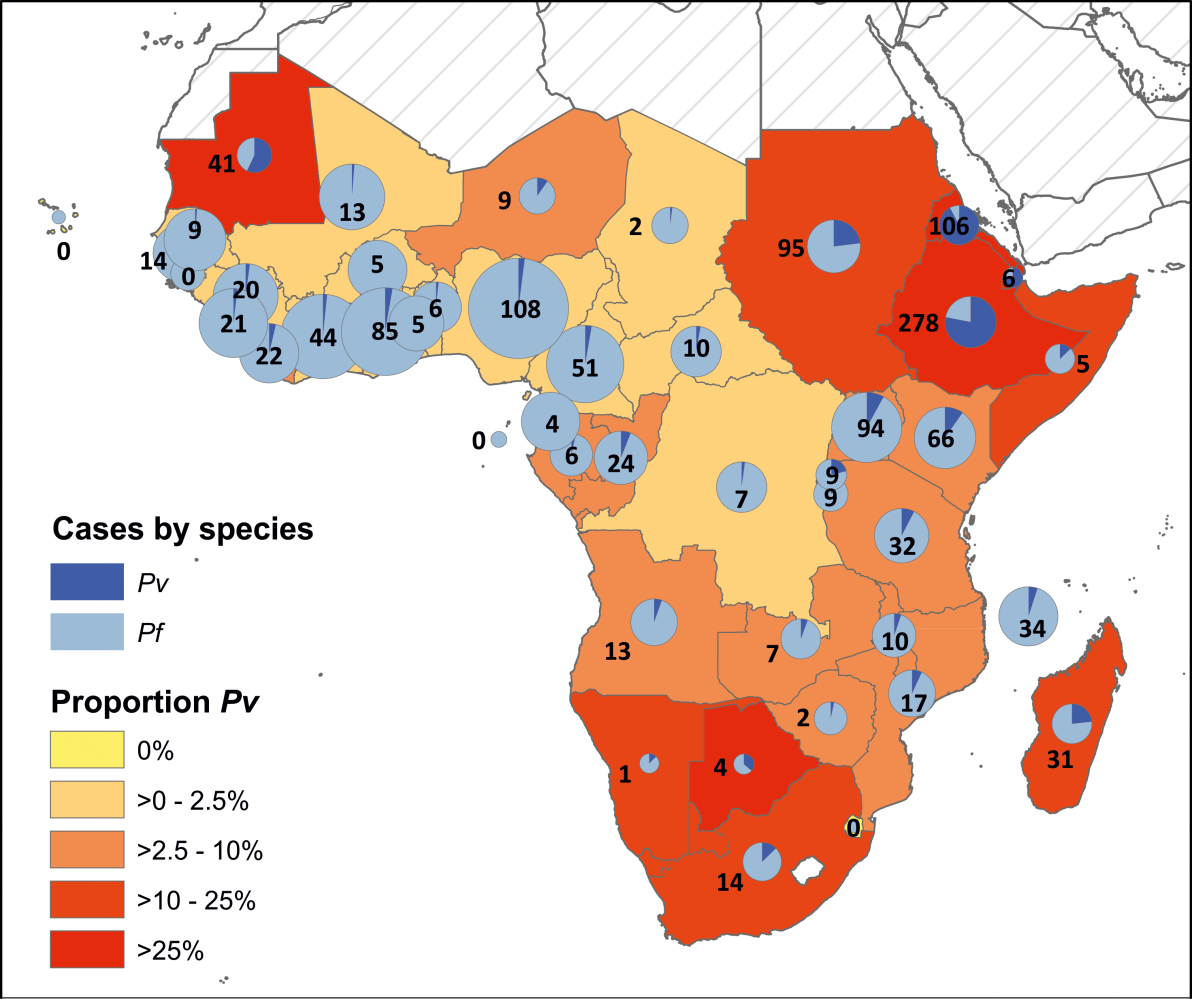
Traveller infections mapped to probable country of infection. The relative contribution of *Pf* and *Pv* infections is reflected in each pie chart (with charts sized by overall number of infections, transformed on a square-root scale). Numbers indicate the number of exported *Pv* infections identified by this study (corresponding to the dark blue segment of the pie chart). Infections date from 2000 to 2014; data sources and countries of importation are listed in Fig 1. Where species-specific information was available for mixed infections, these were considered to be separate infections, so a single individual would have contributed two or more infections to the total count of malaria parasite infections. Returning patients for whom a single country of probable infection could not be determined were excluded.

Records of *Plasmodium* infections in travellers were accessed for 31 malaria-free countries, corresponding to a period of 182 surveillance-years between 2000 and 2014 (Fig 1). Overall, 1,339 *Pv* infections were reported from 42 suspected countries of infection spread across sub-Saharan African countries, corresponding to all but five countries considered here. Fewer than 20 cases of *Pv* were reported from 24 countries, while from nine countries there were 20–50 cases, and nine countries were associated with infection of 50 of more travellers over the reporting period. The number of cases that our searches assembled and the proportion of *Pv* infections relative to *Pf* are shown in Fig 5, though these ratios were not included in the evidence-weighting framework which focussed exclusively on *Pv* with evidence strength classifications based on categorical classifications and not a quantitative analysis. Limitations to this dataset are discussed below.

### Evidence of transmission: The composite map

**Fig 6:**
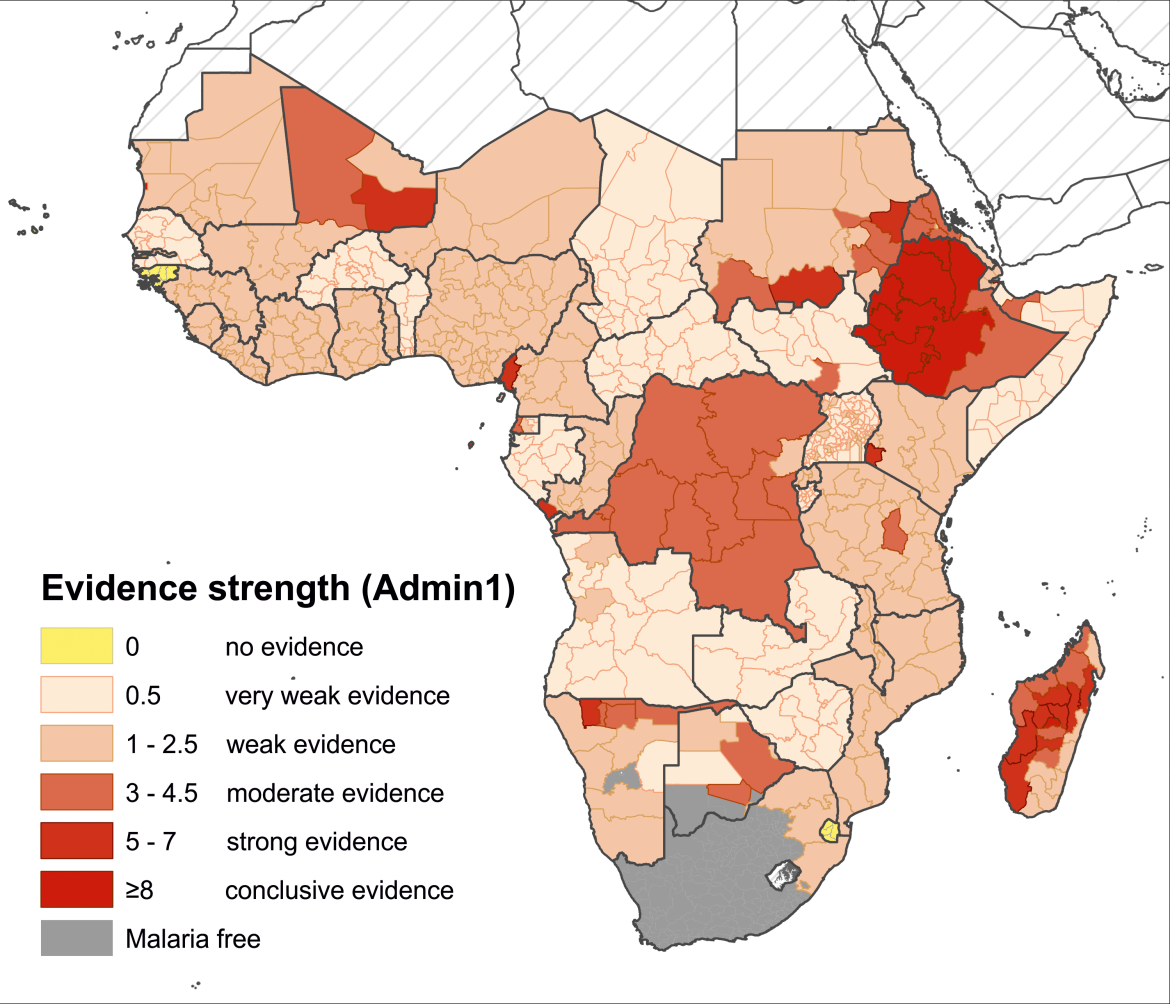
Composite map of the evidence of *Pv* transmission in sub-Saharan Africa, summarised to the Admin1 unit.

The composite map in Fig 6 reflects the strength of the overall available evidence in each Admin1 unit (summarised by country in S5 Table). Of the 629 Admin1 units across malaria-endemic Africa, there were 28 units for which no evidence of transmission was identified (island nations of Cape Verde and Mayotte, and the small states of Guinea-Bissau and Swaziland). In 488 Admin1 units, the only available evidence was from traveller infections, so evidence of transmission from these areas was considered weak (n = 217) or very weak (n = 271). Of the remaining Admin1 units from which direct evidence was identified (n = 113), eight had conclusive evidence (in Ethiopia, Madagascar and Mauritania), 20 had strong, 36 moderate and 49 weak evidence of local transmission. Overall, there was moderate to conclusive evidence of *Pv* from 18 countries, including four in West Africa, three in Central Africa, three in East Africa, two in Southern Africa, and all six countries of the “Horn of Africa+” (HoA+) region (which included Djibouti, Eritrea, Ethiopia, Somalia, Sudan and South Sudan). The weaker evidence categories represent areas of relatively higher uncertainty.

### Population at risk of infection

An estimated 86.6 million individuals were at risk of *Pv* infection (*Pv*PAR) across sub-Saharan Africa in 2015 (interquartile range, IQR: 43.9–156.7 million; S6 Table). These individuals were Duffy positive hosts living outside urban areas across malaria-endemic Africa where temperatures were suitable for sporogony at some point during an average year. The confidence interval is based only on the IQR of the Duffy positive frequency surface [4], without accounting for uncertainty in the population density dataset or the temperature suitability surface. The binary adjustment excluding risk of urban transmission results in a conservative *Pv*PAR estimate which is also not represented in the confidence interval.

**Fig 7:**
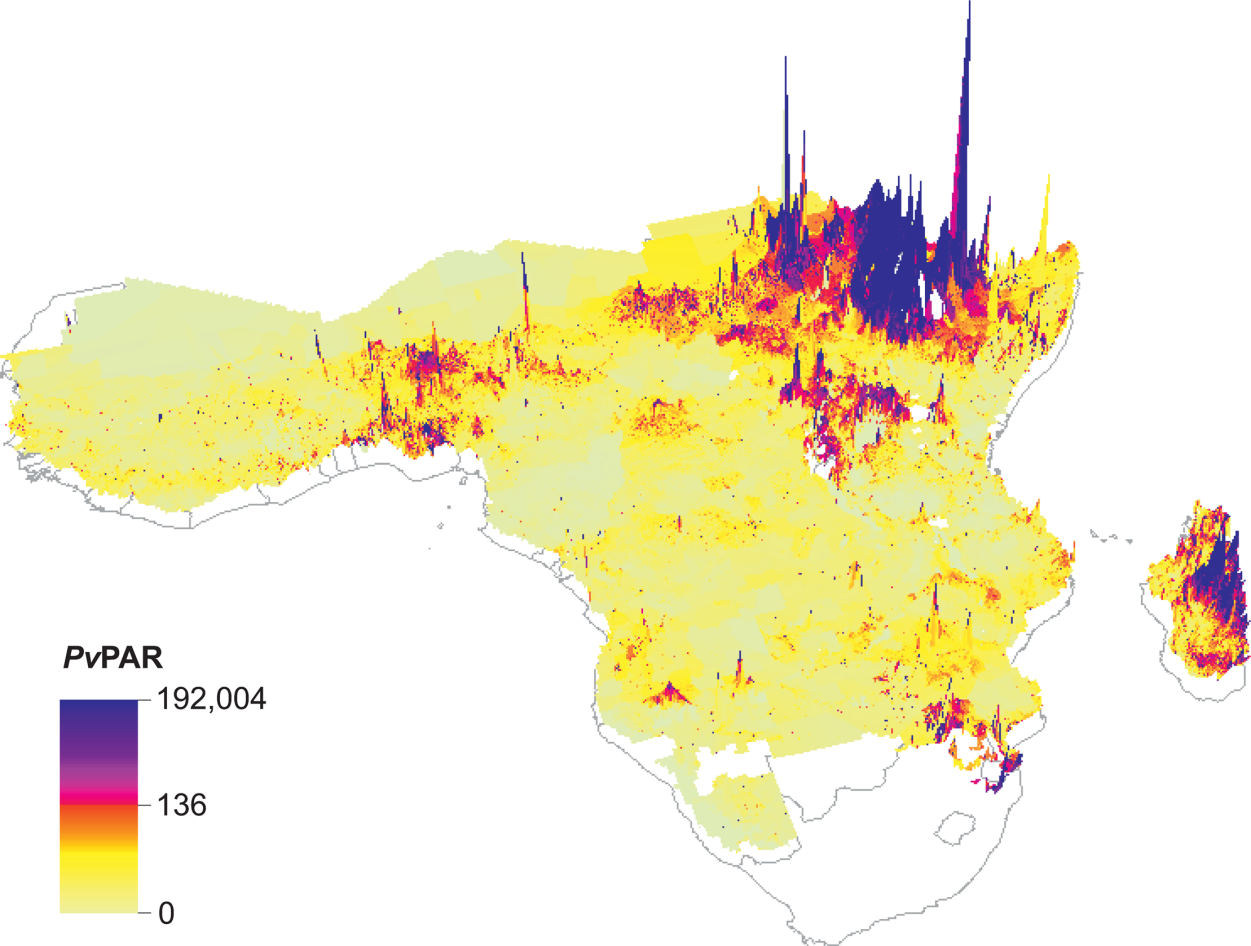
Spatial map of the distribution of the *Pv*PAR. The map resolution is 5 x 5 km, and the 3-dimensional scale was square-root transformed to allow plotting.

Overall, 28% of the *Pv*PAR was outside the HoA+ region, with 10.0 million in East Africa (4.7 million excluding Madagascar), 7.2 million in West Africa, 4.1 million in Southern Africa and 3.3 million in Central Africa (S6 Table). In 12 countries distributed across all sub-regions, the national *Pv*PAR was greater than one million; Ethiopia, Sudan and Madagascar carried the highest *Pv*PARs. The spatial distribution of the *Pv*PAR is illustrated in Fig 7, showing clustered areas of relatively increased *Pv*PAR across the continent.

### Investigation of *Pv*PR in relation the Duffy positive *Pv*PAR and to *Pf*PR

Transmission of *Pv* in sub-Saharan Africa has been considered unlikely given the high prevalence of Duffy negativity across the continent. However, the evidence indicates that *Pv* is present, so here we investigated (i) whether the observed infection rates could be consistent with transmission exclusively limited to Duffy positive hosts, and (ii) how *Pv* and *Pf* infection rates were related to one another in sub-Saharan Africa. Full discussion of the results of these analyses and additional figures are available in the Supplementary Information (S1 Text and S1–S3 Figs).

**Fig 8:**
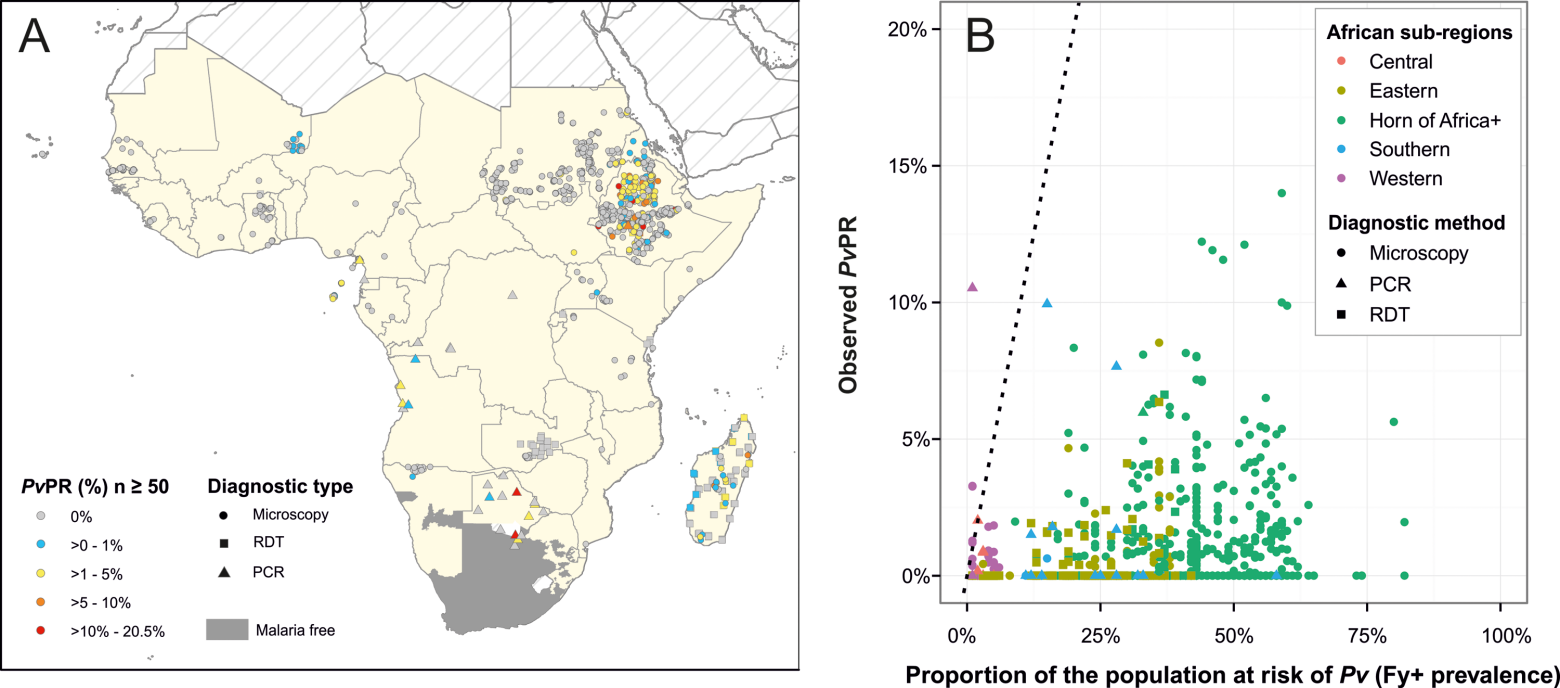
Characteristics of the *Pv*PR dataset (n = 1,546). Only surveys of ≥50 individuals are included, and *Pv*PR values are adjusted to the 1–99 age range. Panel A shows the *Pv*PR values, their spatial distribution and the diagnostic method used for each survey. Panel B is a scatterplot of the relationship between the proportion of the population at risk of *Pv* infection (represented by the proportion of the population Fy+) and the proportion of individuals infected with *Pv* (*Pv*PR).

The data (Fig 8A) were first examined for consistency with transmission within the Duffy positive population subset. The scatter of the data in Fig 8B being almost exclusively concentrated below the *x* = *y* dashed line (1,540 of 1,546 surveys) indicates that the proportion of individuals infected does not exceed the proportion of the population considered susceptible, thus fulfilling the necessary condition (but without proving) that Duffy negativity imposes an upper threshold on *Pv*PR by infections being limited to Duffy positive hosts. Six *Pv*PR values (1.7% of positive surveys) were outliers to this, all from coastal areas of Western/Central regions, including 4 from São Tomé and Príncipe. The survey with the highest discrepancy was from Cameroon, where 13 of 269 individuals were PCR-*Pv* positive [40]. Fig 8B also indicates *Pv*PR rising as the frequency of susceptible hosts increases, as would be expected.

Next, *Pv*PR estimates were re-scaled to prevalence of infection among the subset of Duffy positive hosts (*Pv*PR_Fy+_ = *Pv*PR/Proportion of Duffy positive hosts). S1 Fig plots *Pv*PR_Fy+_ against *Pf*PR, revealing distributions of points across the graphs without any clear trends emerging between infection rates between species. Regional trends, however, were apparent, with higher relative risk of infection in regions of lower host availability. For instance, where *Pv*PR_Fy+_ was positive in the Western region (n = 13), all surveys showed higher likelihood of infection by *Pv* than *Pf* (among the respective susceptible population sub-groups) (S1 and S2 Figs). In areas where Duffy positive hosts were rare (across most of sub-Saharan Africa these are <5%), these individuals were at a higher risk of being infected by *Pv* than by *Pf* (S2 Fig). As Duffy positive hosts become more common, the relative risk of infection becomes more evenly distributed around 0.

Finally, a logistic regression model was developed to test for an association at the population level between the parasite rates of the two species: could PR data from one species predict PR of the other? No significant relationship could be identified from the paired PR dataset for surveys outside the HoA+, and the association was not significantly different from a flat line (S3 Fig). Within the HoA+, *Pf*PR was a highly significant predictor of *Pv*PR_Fy+_ (p = 4.4x10^-6^) up to 15% *Pf*PR, after which increases in *Pf*PR did not result in further predicted increases in *Pv*PR_Fy+_; in contrast, *Pv*PR_Fy+_ within the HoA+ was a significant linear predictor of *Pf*PR at all levels of endemicity. Substantial scatter and heterogeneity in the data, however, resulted in wide 95% confidence intervals around the predicted relationship even when a significant relationship was identified.

### Reports of *P*. *vivax* infection in Duffy negative hosts

**Fig 9:**
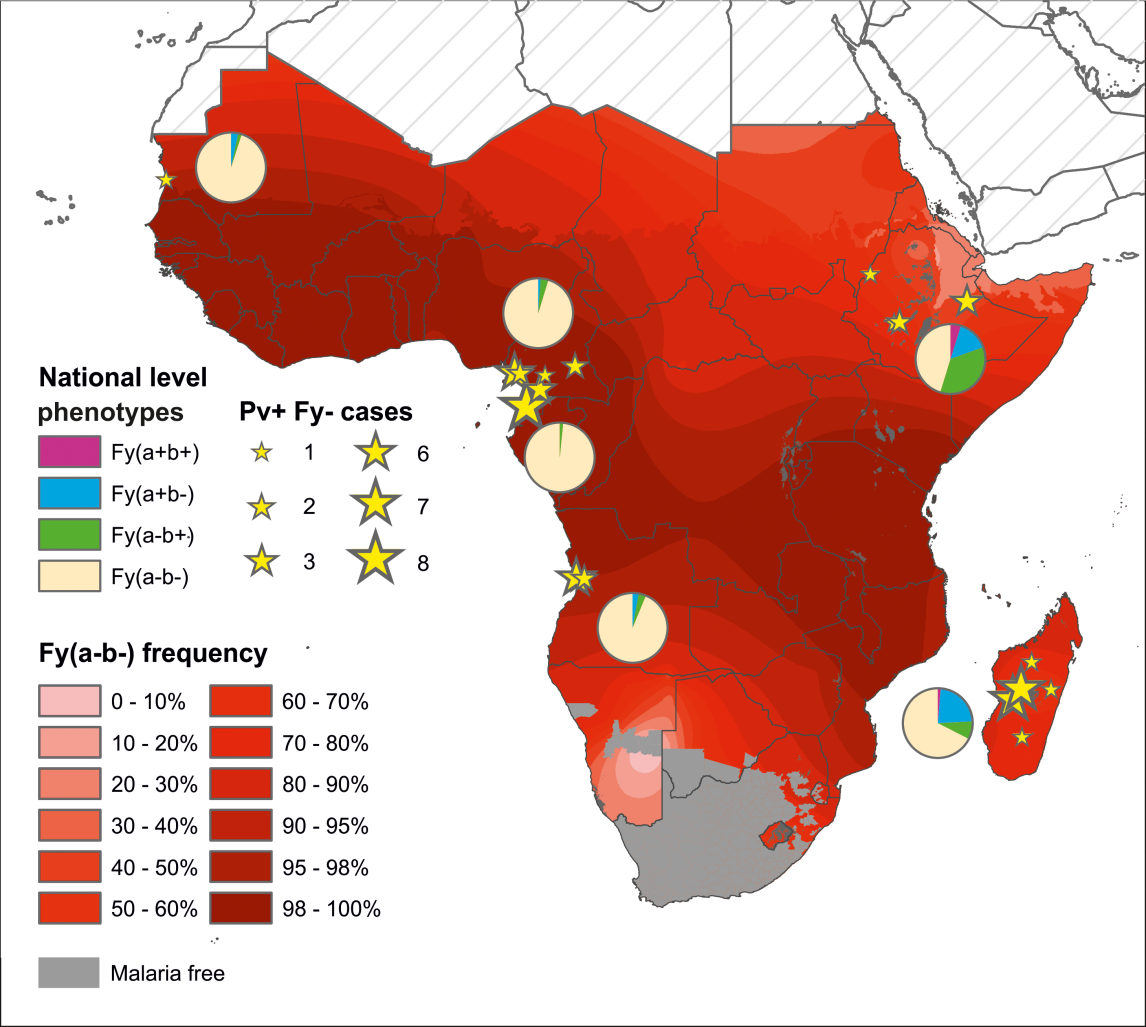
Published observations of molecularly-confirmed *Pv* infections in Duffy negative individuals across Africa (Fy-*Pv*+). Yellow stars represent the number of Fy-*Pv*+ infections identified at each study location (including some as mixed infections with other *Plasmodium* species). Original citations and further details are given in S4 Table. Pie-charts summarise the predicted prevalence of the Duffy phenotypes in each country [41]. The background map is the predicted frequency of Duffy negativity [4]. Further Fy-*Pv*+ infections from western Kenya have been reported [42], but the diagnoses were inconclusive, so are not included in this map.

The last few years have seen evidence emerging of *Pv* infections in Duffy negative hosts (Fy-*Pv*+ infections), providing an additional potential mechanism sustaining *Pv* transmission across sub-Saharan Africa. These observations are from diverse geographic, demographic and host genetic landscapes. The PubMed literature review identified 19 sites across 6 countries of sub-Saharan Africa where Fy-*Pv*+ infections in 54 individuals have been confirmed by molecular diagnosis, all published 2010–2015 (S4 Table and Fig 9). Observations of Fy-*Pv*+ infections were widely distributed across the continent, including in populations with heterogeneous Duffy phenotypes (with correspondingly higher *Pv*PAR: Ethiopia and Madagascar) as well as in populations where Duffy negativity was at near fixation (where *Pv*PAR was much lower: Angola, Cameroon, Equatorial Guinea and Mauritania). All Fy-*Pv*+ infections (except one survey from Yaoundé, Cameroon) were from areas classified as “rural” by GRUMP. The observations were from areas of varied population densities, ranging from <100 to >500,000 individuals in the surrounding 25 km^2^ area (WorldPop Project data). Both symptomatic and asymptomatic Fy-*Pv*+ infections were reported. The reported repartition of *Pv* infections among Duffy negative and positive hosts ranged widely between surveys (S4 Table), but the data did not permit any formal analysis of differential infection risk.

## Discussion

It is evident that *Pv* is not absent from Africa. There is robust evidence from a variety of corroborating sources of *Pv* transmission across all sub-regions, with direct evidence from 21 of the 47 countries considered, and 42 countries are attributed with infection of visiting travellers. Overall, there was moderate to conclusive evidence of *Pv* transmission from within 18 countries. The remaining countries either had only weak evidence of transmission or no evidence could be found at all. These observations of widespread *Pv* infection should not be surprising: a suitable environment, competent vectors and susceptible human hosts make it unlikely that the parasite would not be present. The implications of the present *Pv*-specific study apply to all non-*Pf* species: without adequate diagnosis and reporting, their true public health burden cannot be determined. While *Po* and *Pm* are more widely acknowledged to be endemic to parts of Africa, *Pf*-specific diagnostics will not diagnose the low-level parasitaemia and non-specific symptoms that the non-*Pf* malarias present as, and radical cure is not available for either of the relapsing species, *Pv* or *Po*.

The opportunistic and highly varied nature of the available surveys restricted the sophistication of potential analyses, limiting the composite map to being a qualitative assessment of available evidence rather than providing any quantitative measures or comparisons with other parasite species. For instance, varied molecular diagnostic methodologies or inconsistent approaches for calculating sero-prevalence meant that standardization was not possible even within each data type, therefore favouring the categorical approach. The limited distribution of the data means that the resulting evidence map is both a map of sampling effort and of *Pv* presence in those sampled areas. Greater survey coverage would allow a distancing from the dependency on sampling effort. The traveller data are also biased according to popular travel destinations, and suffer further limitations: (i) the data assembled here are incomplete, formed from an opportunistic selection of countries and reporting years. The numbers of infections cannot be interpreted in any absolute sense; (ii) data are reported only to the national level, whereas the analysis was sub-national; (iii) suspected countries of infection may not be fully reliable given *Pv*’s ability to form latent infections. In recognition of these limitations, traveller data were therefore strongly down-weighted relative to other data types but nevertheless represented an important and complimentary component of the evidence of *Pv* transmission.

Although there were additional surveys reporting “zero *Pv*”, these were not incorporated into the evidence synthesis as they could not be interpreted as “absolute absences” [43], particularly given the lower sensitivity of conventional microscopy and RDT methods to *Pv* than *Pf* [27,29] which often underestimate the true prevalence of blood-stage *Pv* [44–48], especially in mixed infections [49]. The low anticipated prevalence of *Pv* means that very large sample sizes and diagnostic tools with higher sensitivity for detecting *Pv* would be needed to reliably give any confidence of “absence”.

While *Pv* infection levels appear plausible with transmission being restricted to the Duffy positive population at risk (estimated at 86.6 million), a number of other explanations discussed here have also been hypothesised to explain the observed infections.

### Transmission among Duffy positive hosts

The PR data were found to be consistent with transmission being potentially sustained by Duffy positive hosts alone. However, an important limitation to the PR comparative analysis was the skewed distribution of positive surveys, with the majority coming from the HoA+ (73%), while the main area of interest was outside this region. The lack of any predictable association between *Pv*PR_Fy+_ and *Pf*PR in regions outside the HoA+ suggests that change in the prevalence of one species is not reflected by the prevalence of the other species at the host population level. This outcome may indicate that the two infection rates are determined by different drivers of transmission linked to their differing biological characteristics, notably capacity to relapse. For instance in these areas of low Duffy positive host availability, the environmental drivers that are highly significant determinants of *Pf*PR endemicity [50], may be secondary to the impact of the local host dynamics for predicting *Pv*PR_Fy+_. Alternatively, the noise from the heterogeneity of the small dataset (exacerbated by the *Pv*PR adjustment to *Pv*PR_Fy+_) may be masking a relationship similar to that in the more data-rich HoA+ region which would be possible to detect through a larger dataset.

Bespoke transmission models accounting for relapse risks and the peculiarities of the predominantly Duffy negative landscape are required in order to estimate the critical community sizes and determine the transmission dynamics needed to sustain infection, to verify the plausibility of *Pv* in Africa being limited to Duffy positive individuals [51–56].

The quantitative PR data analysis presented here is heavily contingent on the Duffy negativity frequency map [4]. Of the 203 population surveys which informed that map, 41% were published pre-1990. The increase in large-scale population movement since this time [57,58] means that an influx of Duffy positive alleles to the gene pool may be increasing the frequency of susceptible hosts with a strongly spatially clustered distribution, making the 86.6 million *Pv*PAR estimate a potentially significant underestimate. Furthermore, the historical waves of migration from Duffy positive British, French, Indian, Lebanese, Portuguese etc, populations may also be underrepresented. The wide confidence interval of the *Pv*PAR (43.9–156.7 million) indicates the potential impact of changes to the Duffy maps. Furthermore, the GRUMP map which was used to identify urban populations for exclusion from the *Pv*PAR, overestimate urban areas, so many high density rural populations may have been excluded, further contributing to a conservative *Pv*PAR estimate.

### *Plasmodium ovale* misdiagnoses

*Plasmodium ovale* represents an important potential confounder to the reliability of microscopic reports of *Pv* due to their morphological similarities [24] and the risk of *Pv* reports actually being misdiagnosed *Po* infections. Conversely, the opposite may also be true with *Pv* infections being misclassified as *Po* due to the belief of *Pv* absence [3]. Microscopy-based diagnoses were down-weighted relative to PCR-based data in the evidence framework to account for these uncertainties. There is also potential for cross-reactivity between *Po*- and *Pv*-specific antigens in serological screening. Little is known about the diversity of *Po* in particular, so there is a need for the development of additional species-specific reagents to reliably distinguish the two species.

The finding of *Pv* being a common cause of traveller infections is consistently reported from varied sources (Fig 5). For instance, of 618 cases of *Pv* imported into Europe between 1999 and 2003, 33.8% were reported to have been infected in Africa [59]. While molecular confirmation is not routine for returning traveller infections, this is increasingly common in China where thorough case investigations differentiate imported from indigenous cases [60]. Recent investigation of malaria infections among gold miners returning to China from Ghana after a median travel time of one year, found 42 of 874 infections were *Pv* and 1 was *Po* [61]. From a public health perspective, even if these several hundreds of infections are all misdiagnoses, given that the same treatment guidelines for radical cure apply to both species [30], the potential misdiagnosis is not significant in terms of treatment policy and requires the same increased diagnostic capacity away from the perception of *Pf* exclusivity.

### Relapse as a confounder

A potential confounder to attributing all the assembled evidence of *Pv* to local transmission in Africa is the parasite’s ability to form dormant hypnozoites and present clinical symptoms only after a delayed period during which the patient may have travelled widely [14,62]. The evidence-weighting framework attempted to account for this possibility through the differing thresholds between categories. Again, though, even if a subset of the observed cases resulted from infection events elsewhere, the implications for diagnostic and treatment policy remain the same: appropriate capacity is required in the area of clinical presentation, irrespective of where transmission occurred.

### Zoonotic transfer from an ape reservoir

Liu *et al* [63] have published conclusive evidence of the origin of human *Pv* being from a genetically diverse parasite population whose natural hosts are gorillas and chimpanzees in Africa. Although the human parasite clade is distinct from the more diverse parasite strains that indiscriminately infect various ape species, there is evidence (from one individual) of the plausibility of cross-infection of parasite strains between ape and human hosts with similar clinical presentation [63,64]. Liu *et al* therefore argue that *Pv* infections reported from regions of high Duffy negativity frequency are zoonotic infections spilling over from ape reservoirs. Entomological evidence suggests that *An*. *moucheti* and *An*. *vinckei* may be potential vector species bridging transmission between apes and humans [64,65], although the entomological evidence is very limited with only one reported infected specimen of each species and vector behaviour that is not conducive to frequent transmission events.

**Fig 10:**
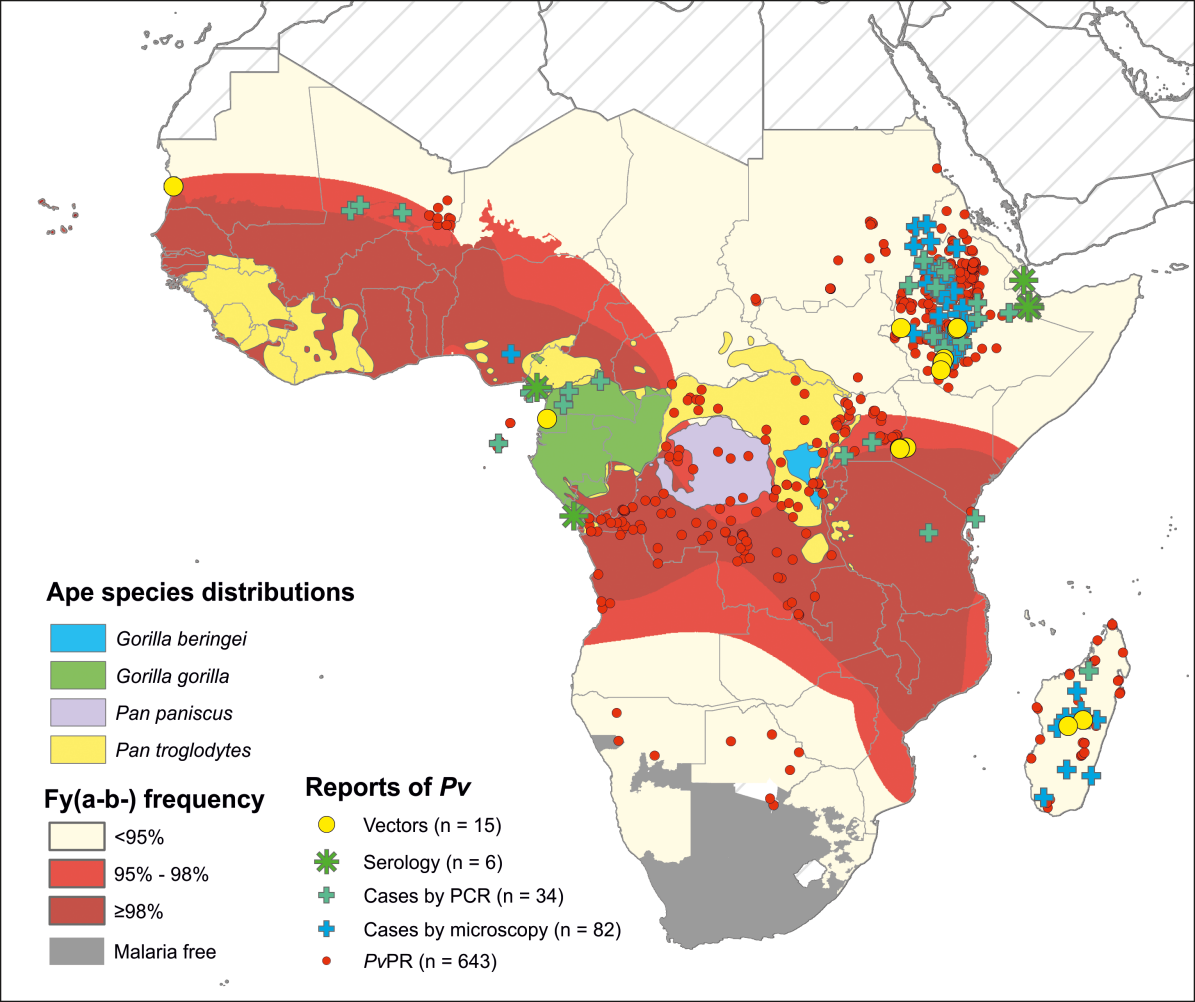
Observations of *Pv* across Africa in relation to the distributions of hypothesised ape reservoir hosts [63] (based on the IUCN Red List of Threatened Species distribution maps [66]) and regions of highest frequencies of Duffy negativity (Fy(a-b-)) [4].

The natural ranges of these ape reservoir species are restricted to the forests of Central Africa (Fig 10). So, while this infection mechanism may explain transmission in specific contexts, this does not represent a universal explanation for the evidence of *Pv* across predominantly Duffy negative regions of Africa.

### Duffy-independent transmission

The reports of Duffy negative infections from diverse epidemiological settings (54 molecularly-confirmed symptomatic and asymptomatic infections identified across 19 sites; S4 Table) represent a further potential mechanism sustaining *Pv* transmission across Africa. The public health burden of these infections is unclear. While the protective effect of Duffy negativity may not be as absolute as previously considered, this blood group’s high frequencies across Africa do nevertheless appear to represent a limiting force on potential *Pv* endemicity. In the absence of any limiting factor, and given the presence of competent vectors and a suitable climate, rates of *Pv* infection comparable to those in Asia or the Americas [31] would be expected. Fy-*Pv*+ infections demand further investigation, ideally across diverse epidemiological settings. The impact of reduced protection conferred by Duffy negativity (currently assumed to be 100%) would have important impacts on the resulting *Pv*PAR (see estimates in Zimmerman *et al* [67]).

### Complementary transmission mechanisms across a highly diverse continent

For the first time, this paper synthesises all available evidence of *Pv* transmission in Africa, and evaluates the varied mechanisms hypothesised to explain these observations across an area where transmission is largely unexpected. Here we argue that no single theory is sufficient to explain transmission across this varied continent, but instead, transmission pathways vary according to the complex ecology and epidemiology of each area and population.

Evidence relating to *Pv* transmission across Africa appears inconsistent. *Pv* is clearly widely present, causing a substantial proportion of returning traveller malaria infections, clinical illness and asymptomatic infection among local residents, infecting vectors and presenting a history of exposure by local communities to infected bites. However, extensive surveys using high-sensitivity molecular methods have repeatedly failed to diagnose *Pv* [68–70]. For instance, PCR-based screening of 1,402 blood samples in southern Cameroon found no trace of *Pv* [69], while a nearby PCR-based community survey of 269 individuals diagnosed a prevalence rate of 5% *Pv*PR (representing 13.8% of all *Plasmodium* infections) [40]. The key difference between these neighbouring surveys in southern Cameroon was the demographic composition of the sampled populations: while no *Pv* was found in the remote, rural village communities [69], the multi-ethnic and highly cosmopolitan population did have *Pv* [40].

While there is some evidence of an ape reservoir enabling zoonotic infections, this does not preclude the possibility of endemic transmission. Fig 10 makes evident the observations of *Pv* outside these ape natural ranges, and furthermore, the main group of travellers returning from Africa with *Pv* infections are visiting relatives [71,72], and less likely to visit the tourist attractions which would bring them into contact with infected vectors [73]. Therefore, while there is evidence of the zoonotic reservoir being a contributing source of infection, other mechanisms of transmission (and reservoirs of infection) must also be involved.

The multiple theories discussed so far for explaining the observations of *Pv* in Africa are not mutually exclusive. Instead, given the limitations to each hypothesis for explaining the overall diversity of transmission, it seems probable that all are involved to some degree. The dominant source of infection would vary between location: in remote forest areas, infections may be from the ape reservoir [64], while in more cosmopolitan areas, transmission may be being sustained by an admixed Duffy positive population. The early production of gametocytes in infections [20] means that even where the host has developed immunity to symptomatic blood-stage parasitaemia from prior exposure, short-lived sub-clinical infections could still be helping to sustain transmission. It is likely that some reported cases of *Pv* are in fact *Po* misdiagnoses, but the sheer abundance of data from various diagnostic methods means this cannot be universal. Similarly, while there is convincing evidence of Fy-*Pv*+ infections, these are not reflected by the widespread *Pv* endemicity levels that would be expected if these infections were commonplace. The epidemiology of *Pv* in Africa is likely to be complex and multi-faceted, driven by different mechanisms in different regions, determined by the unique host genetic, vector, and reservoir characteristics of each area. These complimentary theories together may explain the observed evidence of *Pv* transmission. High sensitivity molecular methods could allow further insight into the population genetics of *Pv* infections diagnosed in Africa and therefore their epidemiology [74], as well as helping to define true exposure and risk of exposure to infection.

### Public health implications of widespread *P*. *vivax* infection risk

The non-specific clinical presentation of malaria means that diagnosis is only possible with a parasitological test [30]. The WHO T3 policy to “Test, Treat, Track” all cases of malaria is very logical in an era of resistance emergence and falling endemicity in many areas [75]. However, it becomes harmful if the “Test” component is not capable of detecting all potential parasites. In the many African countries exclusively reliant on HRP2-based RDT diagnosis (see Box 1), *Pv*, *Po* and *Pm* infected patients who present in clinics with fever symptoms will test negative and could leave untreated. The patients will persist in the community as sources of onward transmission, and, in the case of *Pv* and *Po*, be susceptible to the cumulative impact of clinical relapses.

In turn, therapeutic options for *Pv* are inadequate. While *Pf*-targeted control will treat *Pv* clinical symptoms, it will not impact on the parasite reservoir sustaining the observed blood-stage clinical infections. Even in terms of blood-stage therapy, drug policy in Ethiopia (ranked fourth largest contributor to *Pv* cases globally [29]) maintains chloroquine as first-line treatment despite reports of resistance [76]. A further complication to delivering *Pv*-specific therapy in Africa is the high prevalence of glucose-6-phosphate dehydrogenase deficiency (G6PDd) [77], causing a potentially dangerous intolerance to primaquine, the only available drug for treating the liver-stage parasites [78]. The same issues apply to *Po* radical cure. It has been hypothesised that the high prevalence of G6PDd may be the result of a selective advantage against malaria [79–81], meaning that G6PDd might be under-represented in *Pv* patients and thus primaquine therapy a lesser concern. However, given *Pv*’s preference for invading reticulocytes–young red cells with highest levels of G6PD enzyme activity–it is unlikely that a relatively mild G6PD variant (such as G6PD^A-^, the predominant African variant [82]), would provide any protective advantage against *Pv* infection.

Finally, the evidence presented here has two main implications for *Pv* mapping. First, where *Pv*PR surveys are available, the infection rates reported are consistent with transmission only among Duffy positive hosts. This therefore supports the approach previously followed of restricting the *Pv*PAR to Duffy positive hosts [8,83] and of using the frequency of Duffy positive hosts as an upper threshold to the maximum potential prevalence of infection [31]. Second, the *Pf*PR surface cannot be used to predict *Pv* infection prevalence in Duffy positive hosts, except potentially in transmission settings up to 15% *Pf*PR in the HoA+ region.

### Conclusions

The implications of this paper should not be misinterpreted. This is not a “call to arms” requiring huge additional resources above and beyond the considerable efforts already ongoing into control of *Pf*. Current epidemiological data overwhelmingly indicates that *Pf* is the predominant malaria pathogen across most of sub-Saharan Africa [29,31,68], and that where present, *Pv* prevalence remains low. Nevertheless, despite the estimated 86.6 million individuals at risk of infection, *Pv* is so drastically neglected across this vast and populous continent that without a commitment to add capacity for *Pv* in routine surveillance and reporting, this minor player will remain invisible and any quantitative burden estimates and evidence-based policy decisions impossible. Broadening the outlook beyond *Pf* would simultaneously also strengthen the evidence base relating to the other neglected endemic species, *Po* and *Pm* and increase access to diagnosis and treatment of these other neglected malaria species.

## Supporting Information

S1 Text Supporting methods and results: Investigation of the *Pv*PR data in relation to host population characteristics and *Pf*PR. (DOCX)

S1 Fig Prevalence of infection by *Pf* (*Pf*PR) in relation to *Pv*PR among Duffy positive hosts (*Pv*PR_Fy+_). Panels A and B show the full dataset (n = 1,546); Panels C and D have surveys reporting zero prevalence of either species excluded (n = 249). Panels A and C represent prevalence 0–1; while B and D provide higher resolution of 0–20% prevalence. The six surveys where *Pv*PR exceeded the proportion of Duffy positive hosts were excluded as anomalies.
(TIF)

S2 Fig Regional summaries and relative differences in *Pv*PR_Fy+_ and *Pf*PR (log transformed). Panel A boxplot summarises differences within regions, while Panel B shows relative risk of infection between species in relation to the proportion of Duffy positive hosts, and Panel C shows the relative infection risk in relation to the density of Duffy positive hosts (n = 249).
(TIF)

S3 Fig Logistic-regression model predictions using PR values of one species to predict those of the other (n = 249). The dependent variable is plotted on the y-axis. The *Pv*PR values are adjusted to infection rates in the subset of Duffy positive hosts (*Pv*PR_Fy+_). The solid line represents a significant model fit, while a dashed line indicates that there is no relationship significantly different from zero.
(TIF)

S1 Table Reports of wild-caught *Pv*-infected mosquito vectors. (NR: not reported.)
(XLSX)

S2 Table Reports of serological studies with positive reports of *Pv*. (NR: not reported.)
(XLSX)

S3 Table Local case reports of *Pv* infection among patients in Africa. (NR: not reported.)
(XLSX)

S4 Table Reports of Duffy negative hosts infected by *Pv*. (NR: not reported.)
(XLSX)

S5 Table Summary Admin1 classifications by country. (NR: not reported.)
(XLSX)

S6 Table National *Pv*PAR estimates. (NR: not reported.)
(XLSX)
