# Supplementary material for: Plasmodium vivax Transmission in Africa
Source: PLoS Negl Trop Dis. 2015 Nov 20;9(11):e0004222. doi: 10.1371/journal.pntd.0004222 (PMC4654493; doi:10.1371/journal.pntd.0004222)
Supplement: S1 Text — (DOCX) [file pntd.0004222.s001.docx]

Supporting Information to:

***Plasmodium vivax* transmission in Africa**

Rosalind E. Howes, Robert C. Reiner, Jr., Katherine E. Battle, Joshua Longbottom, Bonnie Mappin,

Dariya Ordanovich, Andrew J. Tatem, Chris Drakeley, Peter W. Gething, Peter A. Zimmerman, David L. Smith and Simon I. Hay.

The Supporting Information to this article comprises:

Supporting methods and results

- **Text S1**: investigation of the characteristics of the *Pv*PR data in relation to host population characteristics and *Pf*PR.

Supporting figures

- **Fig S1.** Prevalence of infection by *Pf* (*Pf*PR) in relation to *Pv*PR among Duffy positive hosts (*Pv*PR_Fy+_).
- **Fig S2.** Regional summaries and relative differences in *Pv*PR_Fy+_ and *Pf*PR (log transformed).
- **Fig S3.** Logistic-regression model predictions using PR values of one species to predict those of the other (n=249).

Supporting tables which detail the data included in the analysis. NR: not reported.

- **Table S1**. Reports of wild-caught *Pv*-infected mosquito vectors
- **Table S2**. Reports of serological studies with positive reports of *Pv*
- **Table S3**. Local case reports of *Pv* infection among patients in Africa
- **Table S4**. Reports of Duffy negative hosts infected by *Pv*
- **Table S5**. Summary Admin1 classifications by country
- **Table S6**. National *Pv*PAR estimates

**Text S1**

Extended methods: Investigation of reported *Pv*PR in relation to the Duffy positive *Pv*PAR

The PR database was used to investigate aspects of *Pv* transmission across sub-Saharan Africa and compare them to *Pf*. Only surveys with the diagnostic capacity to identify both species were included, ensuring that paired estimates of *Pv*PR and *Pf*PR were matched in space, time, and population sample characteristics. Only surveys of ≥50 individuals were included. Estimates of the prevalence of Duffy negativity at the PR survey locations were extracted from the modelled Duffy group frequency maps [[1](#_ENREF_1)]. A trio of spatially-matched estimates were therefore collated for *Pv*PR, *Pf*PR and Duffy negativity prevalence. All datasets are freely available for download ([www.map.ox.ac.uk](http://www.map.ox.ac.uk)). To overcome the impact of the variable ages examined between surveys, PR values were adjusted to a standardized age range of 1-99 years [[2-4](#_ENREF_2)]. Where used, regional breakdowns were based on the Global Burden of Disease sub-regions [[5](#_ENREF_5)], with the addition of a “Horn of Africa+” (HoA+) region (including Djibouti, Eritrea, Ethiopia, Somalia, Sudan and South Sudan), to reflect the different *Pv* epidemiology [[6](#_ENREF_6)] and Duffy blood types [[1](#_ENREF_1)] in this area.

This dataset (n = 1,546) was used to assess: (i) whether the observed *Pv*PR values were consistent with infections being potentially limited exclusively to Duffy positive hosts, (ii) how the prevalence of infection and relative risk of infection differed between species among their specific subset of known susceptible hosts (based on the assumption that the *Pv*PAR was limited to Duffy positive hosts: *Pv*PR_Fy+_ = *Pv*PR/Proportion of Duffy positive hosts), (iii) the relationship between infection prevalence of the two species, and whether prevalence of one could inform predictions of the other. To address this last point, a quasi-binomial logistic regression using a generalized additive model framework was implemented in R [[7](#_ENREF_7)] using the *mgcv* package [[8](#_ENREF_8),[9](#_ENREF_9)] and informed by pairs of positive *Pf*PR and *Pv*PR_Fy+_ values (PR>0 for both species; n = 249).

Extended results: Investigation of *Pv*PR in relation the Duffy positive *Pv*PAR and to *Pf*PR.

*Pv*PR estimates were re-scaled to prevalence of infection among the subset of Duffy positive hosts (*Pv*PR_Fy+_ = *Pv*PR/Proportion of Duffy positive hosts). Fig S1 plots *Pv*PR_Fy+_ against *Pf*PR, revealing distributions of points across the graphs without any clear trends emerging between infection rates between species. At sites where both species are detected (n=249 surveys; Fig S1C-D), both species showed a clustering of PR values <10% with sporadic prevalence values up to 50% with only five surveys reporting PR values >50% for either species. The general infection rates did not therefore seem to differ substantially between *Pv*PR_Fy+_ and *Pf*PR. Regional trends, however, were apparent, with higher relative risk of infection in regions of lower host availability. For instance, where *Pv*PR_Fy+_ was positive in the Western region (n=13), all surveys showed higher likelihood of infection by *Pv* than *Pf* (among the respective susceptible population sub-groups) (Fig S1-S2). The scatterplots in Fig S2 show how relative risk of infection (*Pv*PR_Fy+_ versus *Pf*PR) varies as a function of the availability

**
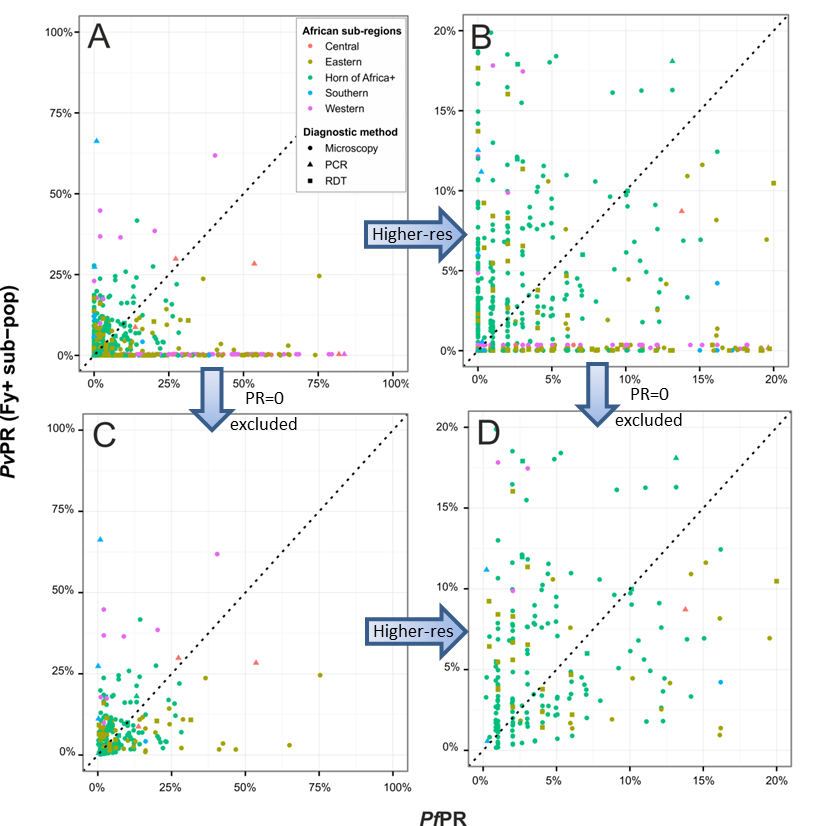
**

**Fig S1. Prevalence of infection by *Pf* (*Pf*PR) in relation to *Pv*PR among Duffy positive hosts (***Pv*PR_Fy+_). Panels A and B show the full dataset (n=1,546); Panels C and D have surveys reporting zero prevalence of either species excluded (n=249). Panels A and C represent prevalence 0-1; while B and D provide higher resolution of 0-20% prevalence. The six surveys where *Pv*PR exceeded the proportion of Duffy positive hosts were excluded as anomalies.

of susceptible hosts for *Pv* infections. In areas where Duffy positive hosts were rare (across most of sub- Saharan Africa these are <5%), these individuals were at a higher risk of being infected by *Pv* than by *Pf* (Fig S2). As Duffy positive hosts become more common (both relatively (Fig S2B) and in absolute number (Fig S2C)), the relative risk of infection becomes more evenly distributed around 0. Finally, a formal framework was used to test whether these trends between species were significant and predictable.


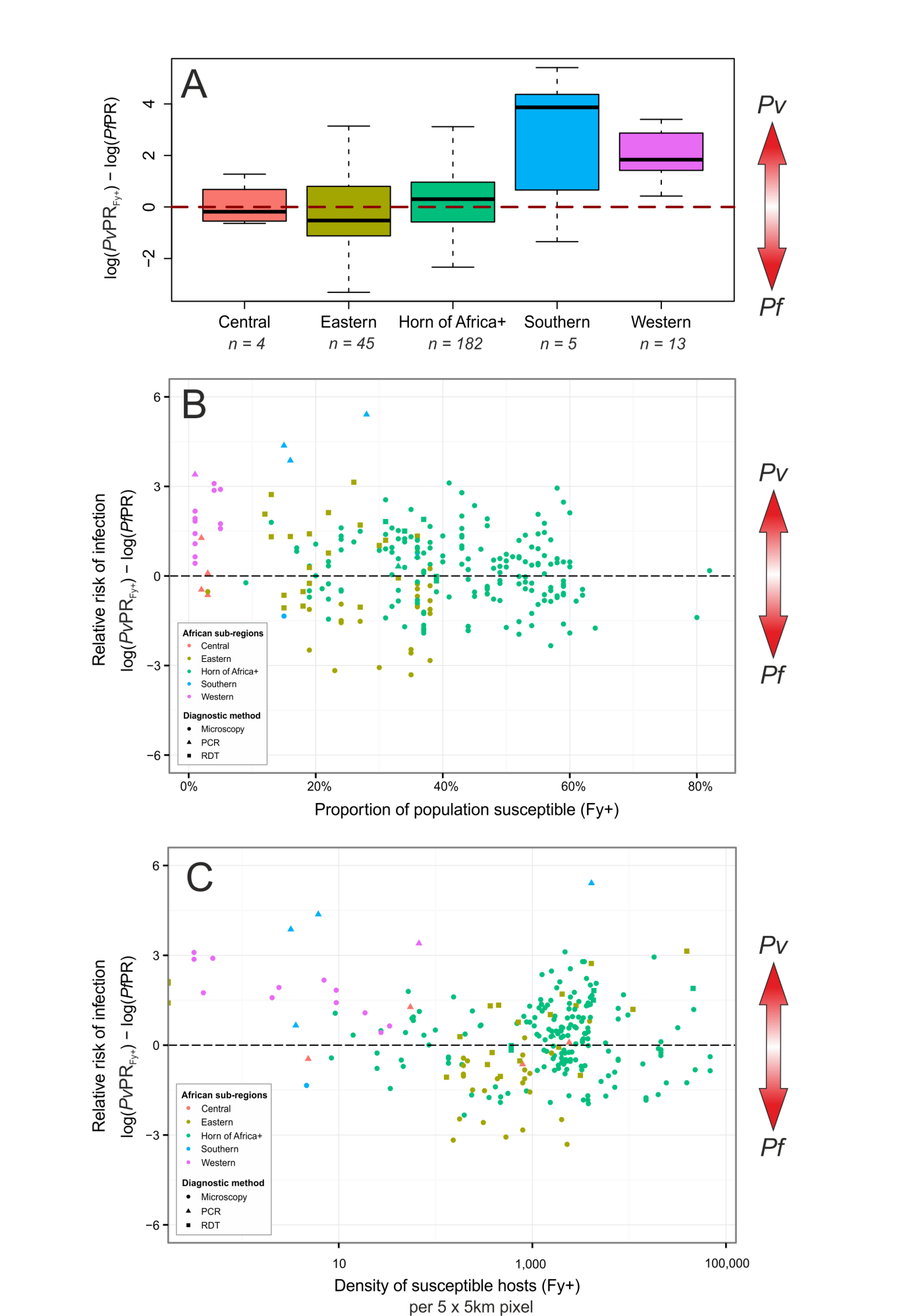


**Fig S2. Regional summaries and relative differences in *Pv*PR_Fy+_** **and *Pf*PR (log transformed).** Panel A boxplot summarises differences within regions, while Panel B shows relative risk of infection between species in relation to the proportion of Duffy positive hosts, and Panel C shows the relative infection risk in relation to the density of Duffy positive hosts (n=249).

A logistic regression model was developed to test for an association at the population level between the parasite rates of the two species: could PR data from one species predict PR of the other? The numbers of surveys available per region were too low to allow meaningful regional analysis, but the HoA+ region (where Duffy phenotypes are heterogeneous and *Pv* is known to be endemic) was modelled separately from the rest of the data. More than double the number of surveys informed the HoA+ model (n=182) versus the other regions combined (n=67). No significant relationship could be identified from the paired PR dataset for surveys outside the HoA+, and the association was not significantly different from a flat line (represented as a dashed line in Fig S3, p=0.357 for *Pf*PR predicting *Pv*PR_Fy+_ and 0.354 for *Pv*PR_Fy+_ predicting *Pf*PR). Within the HoA+, *Pf*PR was a highly significant predictor of *Pv*PR_Fy+_ (p=4.4x10^-6^) up to 15% *Pf*PR, after which increases in *Pf*PR did not result in further predicted increases in *Pv*PR_Fy+_; in contrast, *Pv*PR_Fy+_ within the HoA+ was a significant linear predictor of *Pf*PR at all levels of endemicity. There was substantial scatter and heterogeneity in the data, even where a significant relationship was identified, resulting in wide 95% confidence intervals around the predicted relationship.

**
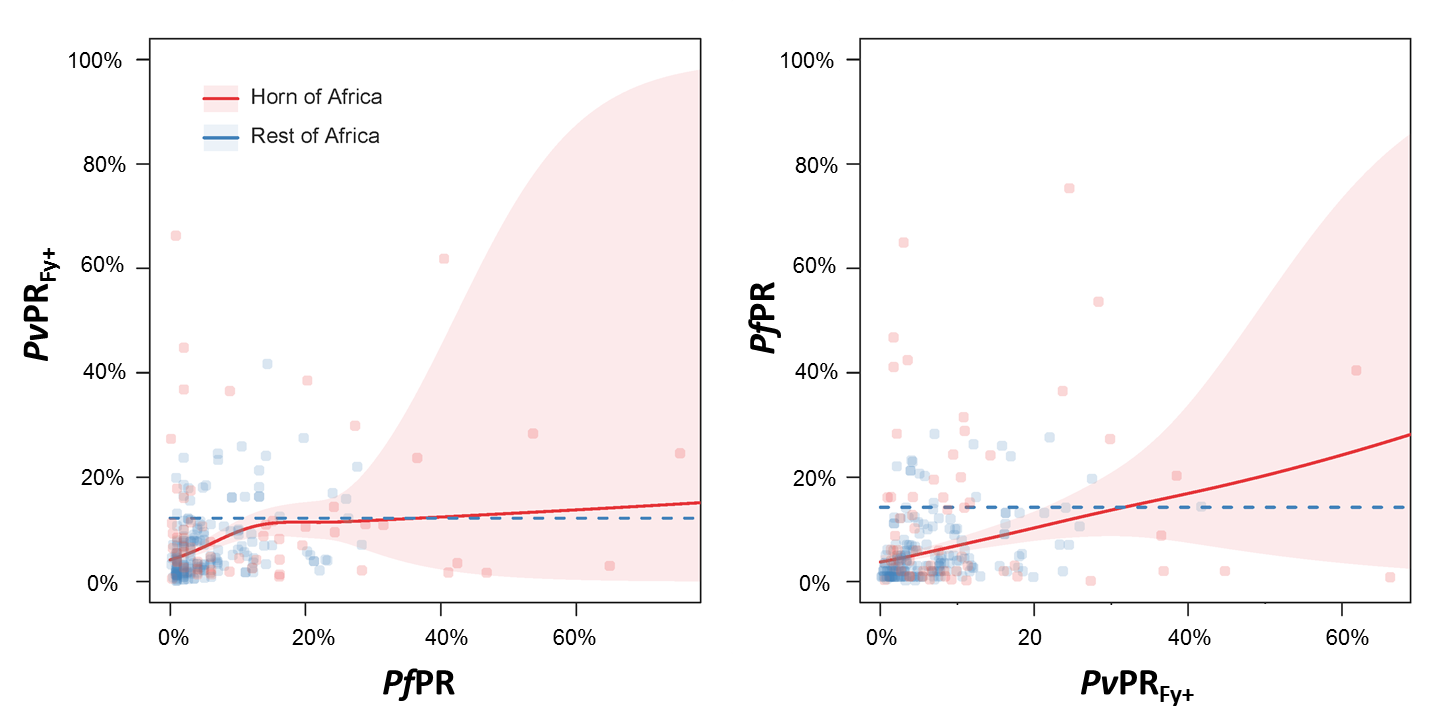
**

**Fig S3. Logistic-regression model predictions using PR values of one species to predict those of the other** (n=249). The dependent variable is plotted on the y-axis. The *Pv*PR values are adjusted to infection rates in the subset of Duffy positive hosts (*Pv*PR_Fy+_). The solid line represents a significant model fit, while a dashed line indicates that there is no relationship significantly different from zero.

**References**

1. Howes RE, Patil AP, Piel FB, Nyangiri OA, Kabaria CW, Gething PW, et al. (2011) The global distribution of the Duffy blood group. Nat Commun 2: 266.

2. Smith DL, Guerra CA, Snow RW, Hay SI (2007) Standardizing estimates of the *Plasmodium falciparum* parasite rate. Malar J 6: 131.

3. Golding N (2014) ageStand R package. GitHub: https://github.com/SEEG-Oxford.

4. Gething PW, Elyazar IR, Moyes CL, Smith DL, Battle KE, Guerra CA, et al. (2012) A long neglected world malaria map: *Plasmodium vivax* endemicity in 2010. PLoS Negl Trop Dis 6: e1814.

5. Murray CJ, Ezzati M, Flaxman AD, Lim S, Lozano R, Michaud C, et al. (2012) GBD 2010: design, definitions, and metrics. Lancet 380: 2063-2066.

6. WHO (2014) World Malaria Report 2014. Geneva, Switzerland: World Health Organization.

7. R Core Team (2014) R: A language and environment for statistical computing. Vienna, Austria: R Foundation for Statistical Computing, <http://www.R-project.org>.

8. Wood S (2015) mgcv: mixed GAM computation vehicle with GCV/AIR/REML smoothness estimation (version 1.8-6). <http://cran.r-project.org/web/packages/mgcv/index.html>.

9. Wood SN (2011) Fast stable restricted maximum likelihood and marginal likelihood estimation of semiparametric generalized linear models. Journal of the Royal Statistical Society: Series B (Statistical Methodology) 73: 3-36.
