# Supplementary figures and images for: Plasmodium vivax Transmission in Africa
Source: PLoS Negl Trop Dis. 2015 Nov 20;9(11):e0004222. doi: 10.1371/journal.pntd.0004222 (PMC4654493; doi:10.1371/journal.pntd.0004222)

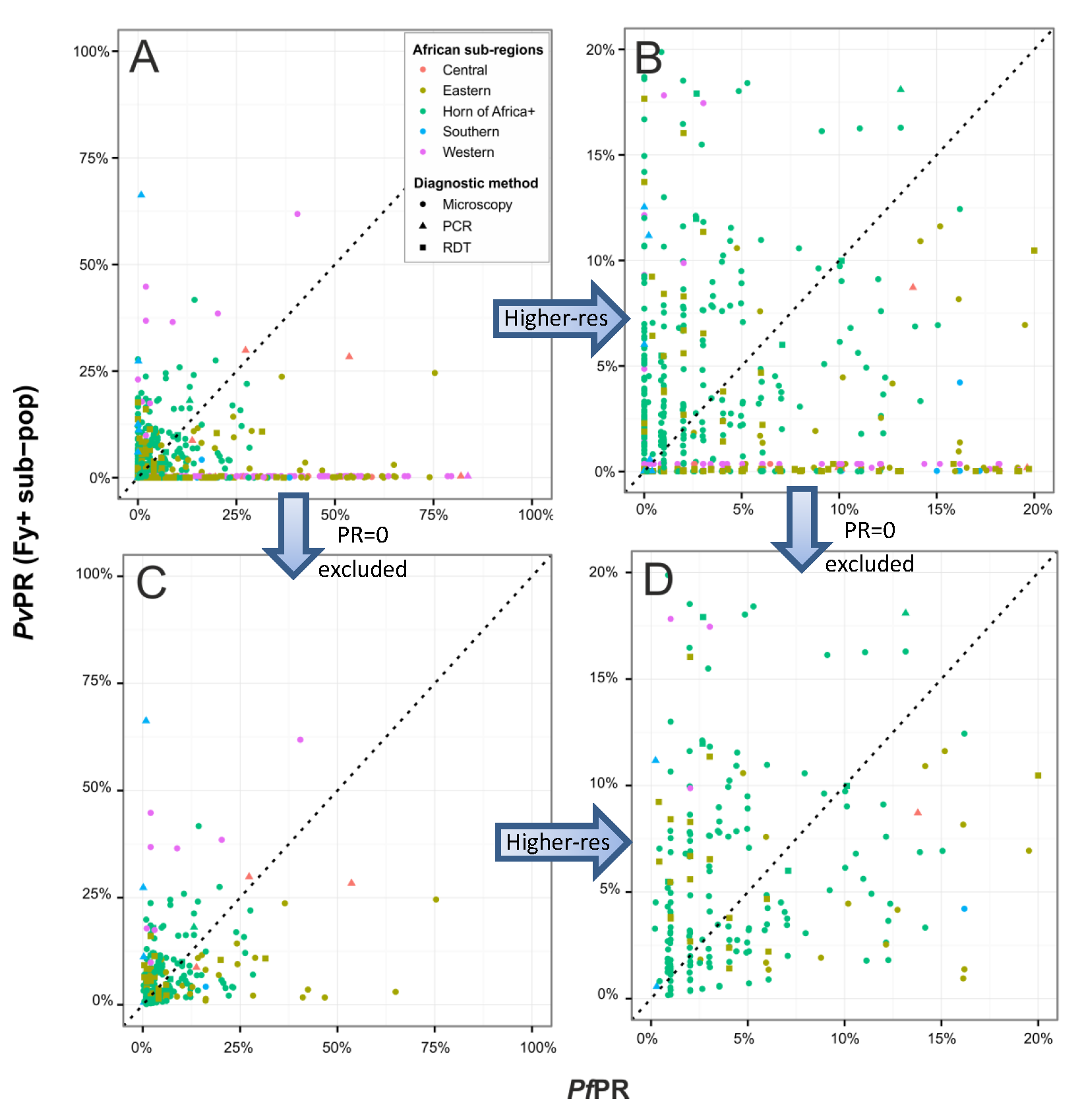

Supplement: S1 Fig — Panels A and B show the full dataset (n = 1,546); Panels C and D have surveys reporting zero prevalence of either species excluded (n = 249). Panels A and C represent prevalence 0–1; while B and D provide higher resolution of 0–20% prevalence. The six surveys where PvPR exceeded the proportion of Duffy positive hosts were excluded as anomalies. (TIF) [file pntd.0004222.s002.tif]

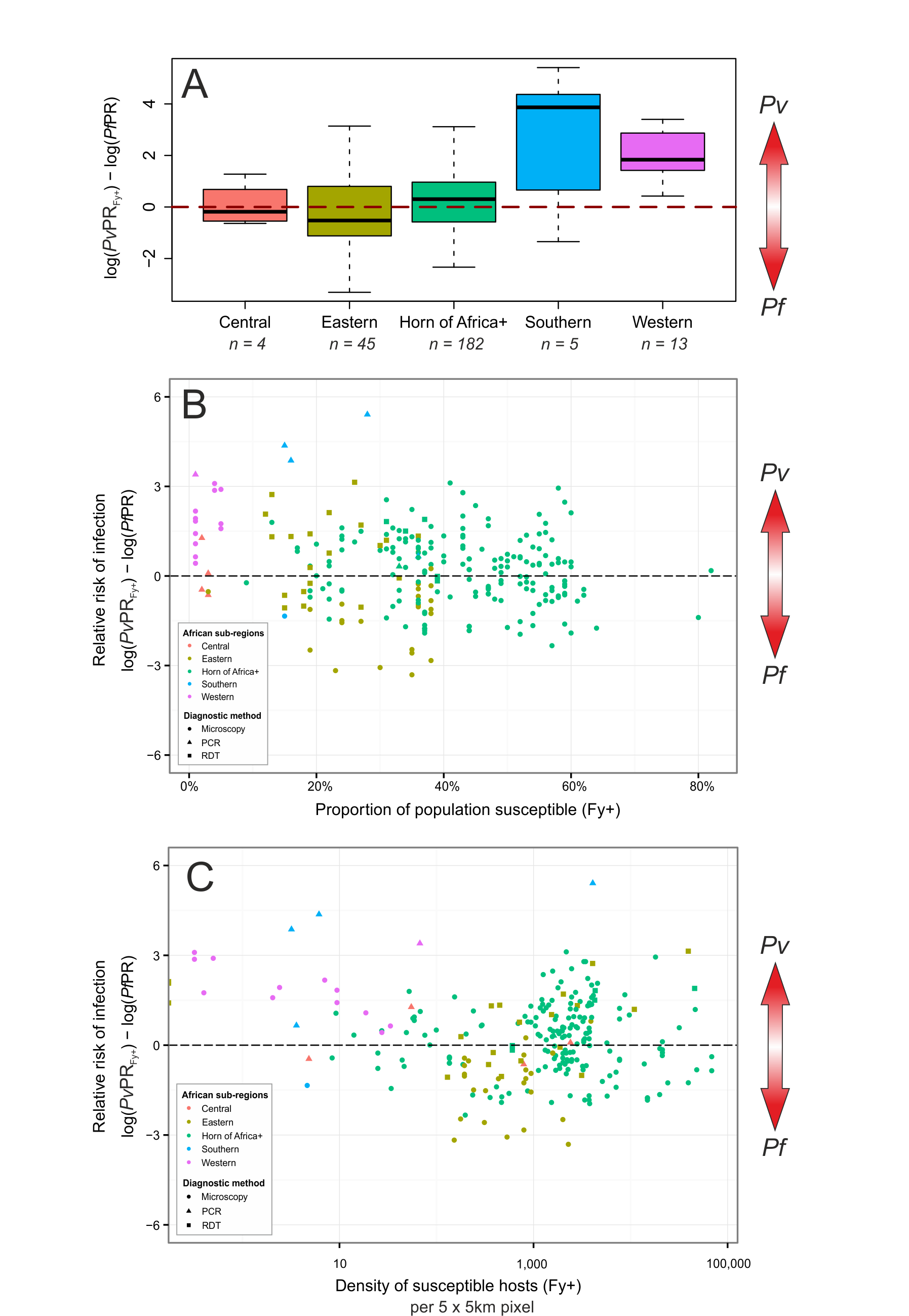

Supplement: S2 Fig — Panel A boxplot summarises differences within regions, while Panel B shows relative risk of infection between species in relation to the proportion of Duffy positive hosts, and Panel C shows the relative infection risk in relation to the density of Duffy positive hosts (n = 249). (TIF) [file pntd.0004222.s003.tif]

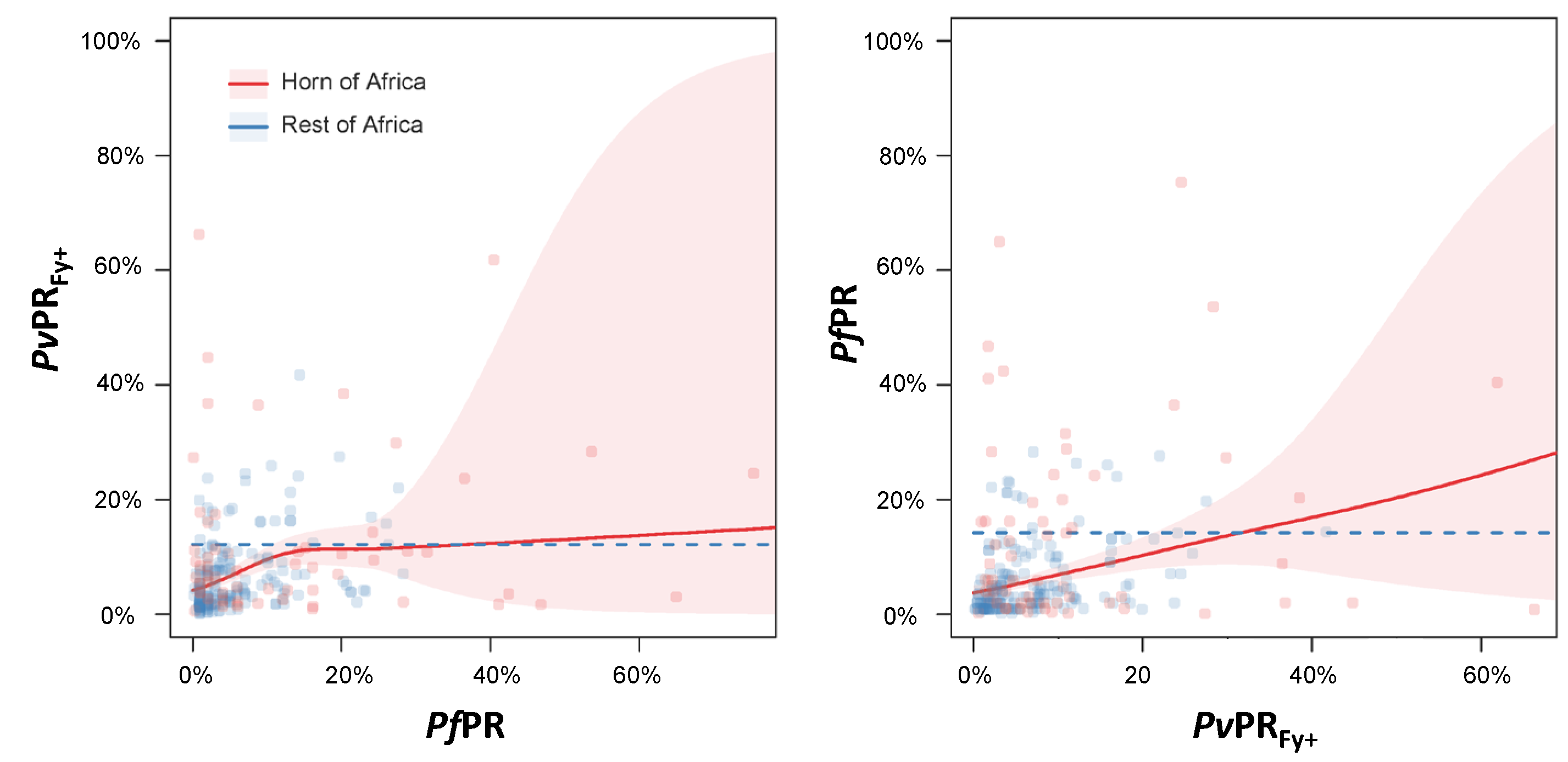

Supplement: S3 Fig — The dependent variable is plotted on the y-axis. The PvPR values are adjusted to infection rates in the subset of Duffy positive hosts (PvPRFy+). The solid line represents a significant model fit, while a dashed line indicates that there is no relationship significantly different from zero. (TIF) [file pntd.0004222.s004.tif]
